# Differential pairing of transmembrane domain GxxxG dimerization motifs defines two HLA-DR MHC class II conformers

**DOI:** 10.1016/j.jbc.2023.104869

**Published:** 2023-05-27

**Authors:** Lisa A. Drake, Amy B. Hahn, Ann M. Dixon, James R. Drake

**Affiliations:** 1 Department of Immunology and Microbial Disease, Albany Medical College, Albany, New York, USA; 2 Transplant Immunology Laboratory, Department of Surgery, Albany Medical College, Albany, New York, USA; 3 Department of Chemistry, University of Warwick, Coventry, United Kingdom

**Keywords:** antigen presentation, major histocompatibility complex (MHC), monoclonal antibody, protein conformation, structural model, HLA, MHC class II

## Abstract

MHC class II molecules function to present exogenous antigen-derived peptides to CD4 T cells to both drive T cell activation and to provide signals back into the class II antigen-presenting cell. Previous work established the presence of multiple GxxxG dimerization motifs within the transmembrane domains of MHC class II α and β chains across a wide range of species and revealed a role for differential GxxxG motif pairing in the formation of two discrete mouse class II conformers with distinct functional properties (*i.e.*, M1-and M2-paired I-A^k^ class II). Biochemical and mutagenesis studies detailed herein extend this model to human class II by identifying an anti-HLA-DR mAb (Tü36) that selectively binds M1-paired HLA-DR molecules. Analysis of the HLA-DR allele reactivity of the Tü36 mAb helped define other HLA-DR residues involved in mAb binding. *In silico* modeling of both TM domain interactions and whole protein structure is consistent with the outcome of biochemical/mutagenesis studies and provides insight into the possible structural differences between the two HLA-DR conformers. Cholesterol depletion studies indicate a role for cholesterol-rich membrane domains in the formation/maintenance of Tü36 mAb reactive DR molecules. Finally, phylogenetic analysis of the amino acid sequences of Tü36-reactive HLA-DR β chains reveals a unique pattern of both Tü36 mAb reactivity and key amino acid polymorphisms. In total, these studies bring the paradigm M1/M2-paired MHC class II molecules to the human HLA-DR molecule and suggest that the functional differences between these conformers defined in mouse class II extend to the human immune system.

A prime function of major histocompatibility complex (MHC) molecules is to present antigen-derived peptides to T lymphocytes to elicit or direct a cellular immune response. MHC class I molecules present peptides derived from cytosolic (*i.e.*, endogenous) antigens to CD8 T cells, whereas MHC class II molecules present peptides derived from endosomal/lysosomal (*i.e.*, exogenous) antigens to CD4 T cells. X-ray crystallography has been used extensively to determine the structure of the extracellular domain of MHC class I and class II molecules and has defined a basic configuration of a membrane-proximal “base” composed of two immunoglobulin-like domains, supporting a β sheet and two helices that form the molecule’s peptide binding groove.

MHC molecules are integral membrane proteins, with transmembrane (TM) and cytosolic domains in addition to the molecule’s extracellular domain, and these domains have numerous immunological functions. The cytoplasmic and TM domains of both MHC class I and class II molecules can mediate signaling within antigen-presenting cells (1, 2, 3) and can impact the association of MHC molecules with other membrane proteins (1, 2). Because MHC class II molecules have two TM domains, differential pairing of the TM domains can impact the structure and function of the entire molecule (4).

From mice to sheep to humans, the TM domains of MHC class II molecules possess multiple highly conserved GxxxG dimerization motifs (5). GxxxG dimerization motifs were originally characterized in glycophorin A and later expanded to be known as GAS_right_ motifs (6) which include the additional small residues Ala and Ser. These small residues facilitate close packing of TM helices and stable van der Waals interactions that can lead to strong TM domain interactions (7, 8). Initial biochemical and molecular modeling studies focused on human class II HLA-DR revealed that a single GxxxG motif in the HLA-DR β chain (*i.e.*, DRB) is able to pair with either of two similar motifs in the HLA-DR α chain (*i.e.*, DRA) (5). Subsequent studies in mouse II I-A^k^ class demonstrated that differential pairing of TM domain GxxxG motifs drives the formation of two different MHC class II conformers that can be distinguished by the 11-5.2 monoclonal antibody (mAb) which binds the molecule’s extracellular domain (9, 10, 11). Specifically, the 11-5.2 mAb selectively recognizes I-A^k^ class II molecules where the single GxxxG motif in the molecule’s β chain associates with the N-terminal M1 GxxxG motif of the I-A^k^ α chain (*i.e.*, M1-paired I-A^k^). While M1-paired mouse class II molecules represent a numerically minor fraction of all cell surface class II molecules, they drive important class II functions such as stimulation of naive T cells (12), formation of B cell-T cell conjugates (9), MHC class II signaling (13, 14), and are selectively loaded with peptides derived from BCR-bound antigen (1, 15). In contrast, the more abundant M2-paired class II molecules can actively block signaling by M1-paired class II (13), and are more readily loaded with peptides derived from non-cognate antigens (1). The roles of M1-and M2-paired class II molecules in the immune response have been recently reviewed (1, 4).

In this report, the fine specificity of the Tü36 mAb that selectively recognizes M1-paired human HLA-DR class II molecules is defined. The binding of the Tü36 mAb is selectively impaired by mutation of the DRA M1 GxxxG motif, and the mAb only immunoprecipitates a fraction of all HLA-DR molecules. Analysis of Tü36 DR allele reactivity defines multiple determinants of antibody recognition, and cholesterol depletion studies reveal a role of lipid raft membrane domains in the formation/maintenance of the Tü36 epitope. Finally, molecular modeling provides insight into both the impact of GxxxG motif association/mutation on TM domain pairing and how TM domain pairing might impact overall molecular structure. The potential implications of these findings are discussed.

## Results

### The Tü36 anti-DR mAb selectively binds M1-paired HLA-DR molecules

Previous work established the unique reactivity of the 11-5.2 mAb toward M1 paired mouse I-A^k^ MHC class II molecules (10, 11). In those reports, the 10-3.6 mAb, which was shown to recognize all I-A^k^ class II molecules irrespective of TM domain pairing, was regularly used as a control in the characterization of the 11-5.2 mAb and I-A^k^ class II conformers (9, 10). To extend the paradigm of M1/M2-paired MHC class II to human HLA-DR class II molecules, the first step was to identify a 10-3.6-like anti-HLA-DR (anti-DR) mAb that binds all HLA-DR molecules. To this goal, the reactivity of the widely used L243 anti-DR mAb was determined/confirmed. L243 binds the highly conserved DRA chain and the L243 epitope requires proper HLA-DR molecule assembly (16). Consistent with this reactivity, L243 has previously been reported to bind all HLA-DR molecules expressed by human T-cell clones (17).

**Figure 1:**
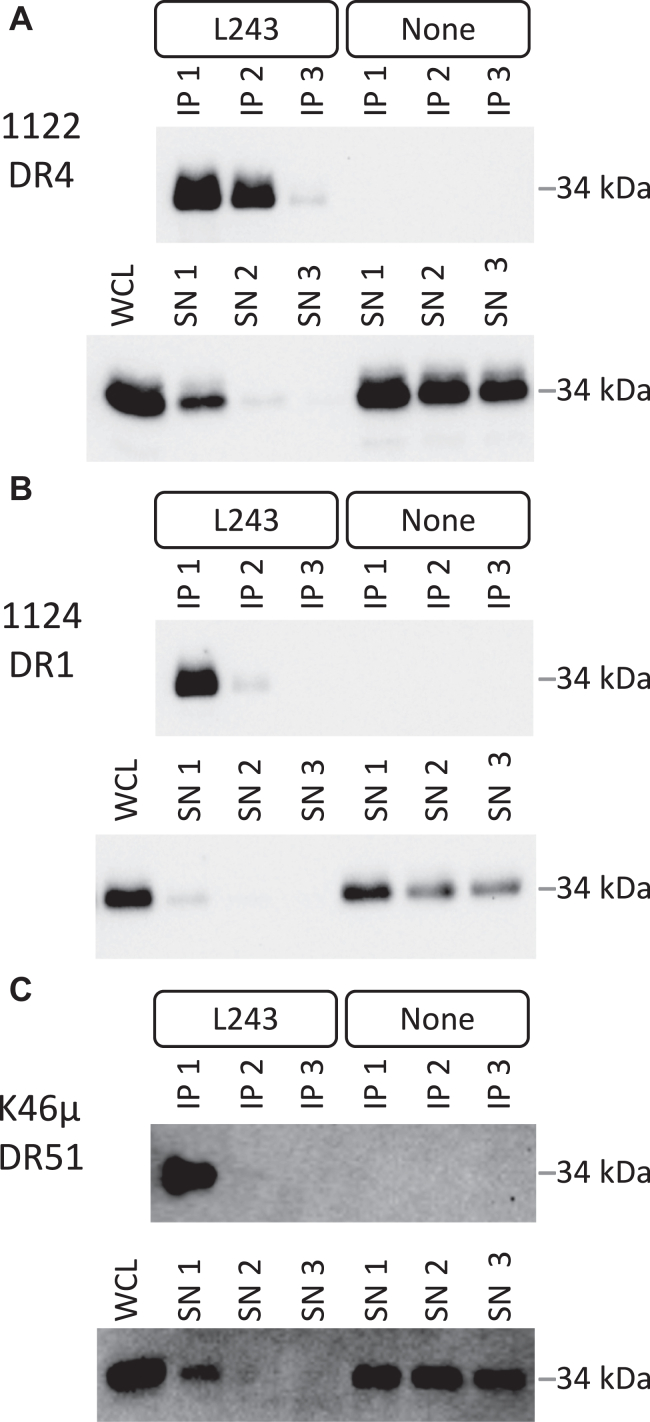
**L243 mAb recognizes all HLA-DR class II molecules.** The indicated cells were lysed in RIPA buffer. Cleared WCL was subjected to three rounds of IP with L243 and Protein A–agarose beads (“L243”). As a control, WCL was IP-ed with just Protein A–agarose (“none”). IP and supernatant (SN) from each round were probed for DRA by Western blot. The *gray bar* to the *right* of each blot indicates the position of the 34 kDa molecular weight standard. Shown are representative results from one of three or more independent experiments for each cell type. IP, immunoprecipitations; WCL, whole cell lysate.

HLA-DR molecules are highly polymorphic. While there are only approximately 40 DRA alleles encoding five different DRA polypeptides, there are over 4200 known DRB alleles encoding over 2800 DRB polypeptide chains (https://www.ebi.ac.uk/ipd/imgt/hla/about/statistics/). For initial analyses of L243 specificity, three simple systems where only a single HLA-DR allele is expressed were used. The first was the 1122 human B cell line expressing only DR4 molecules (DRA∗01:01/DR1∗04:01). L243 mAb was used for multiple sequential immunoprecipitations (IPs) of whole cell lysate (WCL) from 1122 B cells (Fig. 1*A*). For each round of IP, the level of DR in the IP as well as in the IP supernatant (SN) was determined by Western blot for DRA. After the third round of IP, there was no detectable DRA left in the SN indicating that the L243 mAb removes all DR molecules from the WCL, consistent with its reported pan-reactivity (17). Similar results were obtained using WCL from 1124 human B cells expressing DR1 (DRA∗01:01/DRB1∗01:01 – Fig. 1*B*) and from murine K46μ B cells expressing transfected HLA-DR51 molecules (DR51, DRA∗01:02/DRB5∗01:01 – Fig. 1*C*). Taken together, the results confirm that the L243 mAb exhibits broad reactivity for all HLA-DR class II molecules.

**Figure 2:**
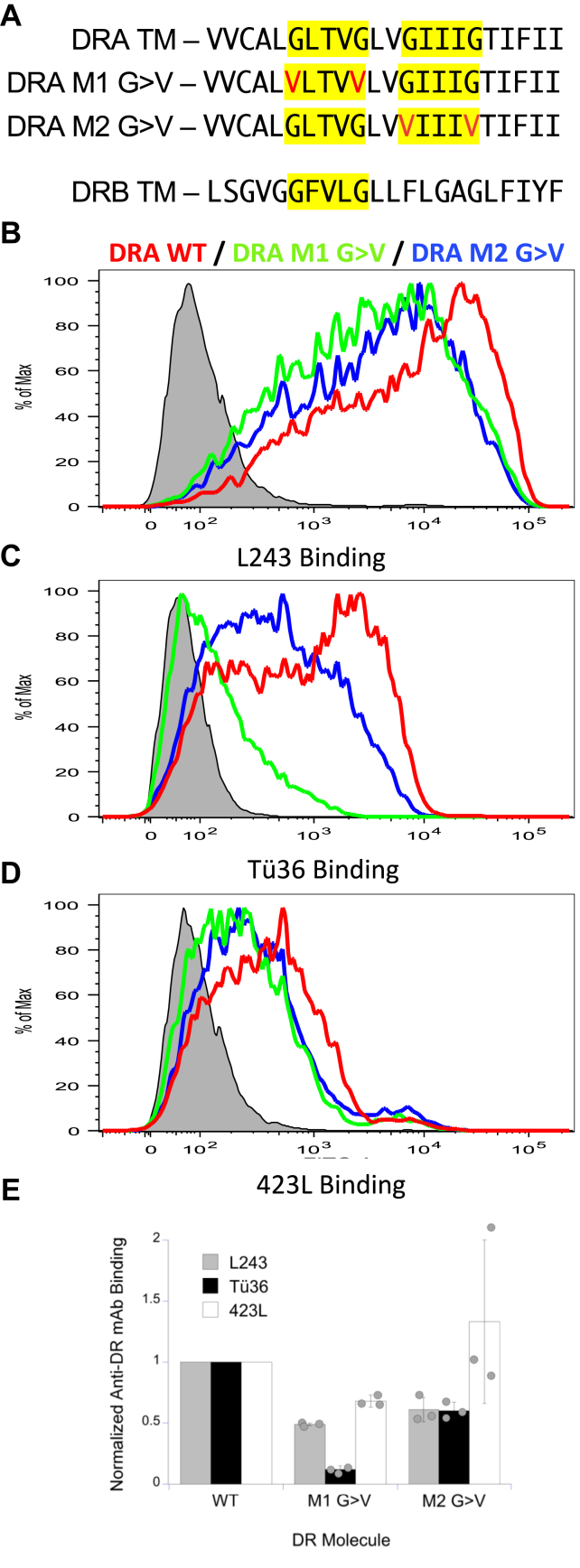
**Mutation of DRA transmembrane domain GxxxG dimerization motif selectively inhibits Tü36 mAb binding.***A*, amino acid sequence of tested DRA TM domains. GxxxG dimerization motifs are highlighted. Mutated amino acid residues are in red. *B*–*D*, 293T cells were transfected with the indicated DRA chains along with DRB1∗04.03. Cells were stained with the indicated anti-DR mAb and analyzed by flow cytometry. The *gray histogram* in each panel illustrates the staining of untransfected 293T cells. *E*, the level of mAb binding across multiple experiments was compared. In each experiment, binding of the indicated mAb to cells expressing WT DR was set to 1.0 and mAb binding to other cells normalized to this value. Bars indicate ±1 SD from across three independent experiments. TM, transmembrane.

To identify anti-DR mAbs that selectively bind to M1 or M2 paired HLA-DR class II molecules, we tested the binding of a panel of anti-HLA-DR mAbs to 293T cells expressing either wild-type (WT) HLA-DR4 (DRA∗01:01/DRB1∗04:01) or HLA-DR4 bearing a DRA chain M1 *or* M2 GxxxG>VxxxV mutation (*i.e.*, DR4 M1 G>V or DR4 M2 G>V, Fig. 2*A*), which will block M1- or M2-based TM domain pairing (5, 10, 11). The binding of the pan-reactive L243 anti-DR mAb was used as a readout of *total* DR expression by each cell population (Fig. 2*B*). Similar to results previously reported in the mouse I-A^k^ system (10, 11), DR4 M1 G>V and DR4 M2 G>V molecules are expressed at levels slightly lower than WT DR4 (Fig. 2*B*). Staining of a sample of the same preparation of cells with the Tü36 anti-DR mAb (18) gives an interesting pattern of labeling (Fig. 2*C*). Cells expressing WT DR4 are stained robustly by Tü36, whereas cells expressing DR4 M1 G>V exhibit very low levels of staining, significantly lower than would be predicted by the L243 staining, (Fig. 2*E*). In contrast, cells expressing DR4 M2 G>V are stained at moderate levels with Tü36 (Fig. 2*C*), in line with what would be predicted based on the L243 staining of these cells (Fig. 2*E*). Staining of the same three preparations of cells with 423L (another broadly-reactive anti-HLA-DR mAb) gives a pattern of staining very similar to L243, albeit at lower levels (Fig. 2, *D* and *E*). These results demonstrate that mutation of the DRA M1 GxxxG motif to VxxxV (which prevents the formation of an M1-paired HLA-DR conformer (10)), selectively impairs DR recognition by the Tü36 mAb, revealing that Tü36 selectively binds M1 paired HLA-DR molecules. Staining of these same cells with the MEM-266 mAb that binds empty HLA-DR molecules (19) fails to reveal any binding, suggesting that failure to bind peptide is not the reason for decreased Tü36 mAb binding to the M1 G>V mutant DR (not shown).

**Figure 3:**
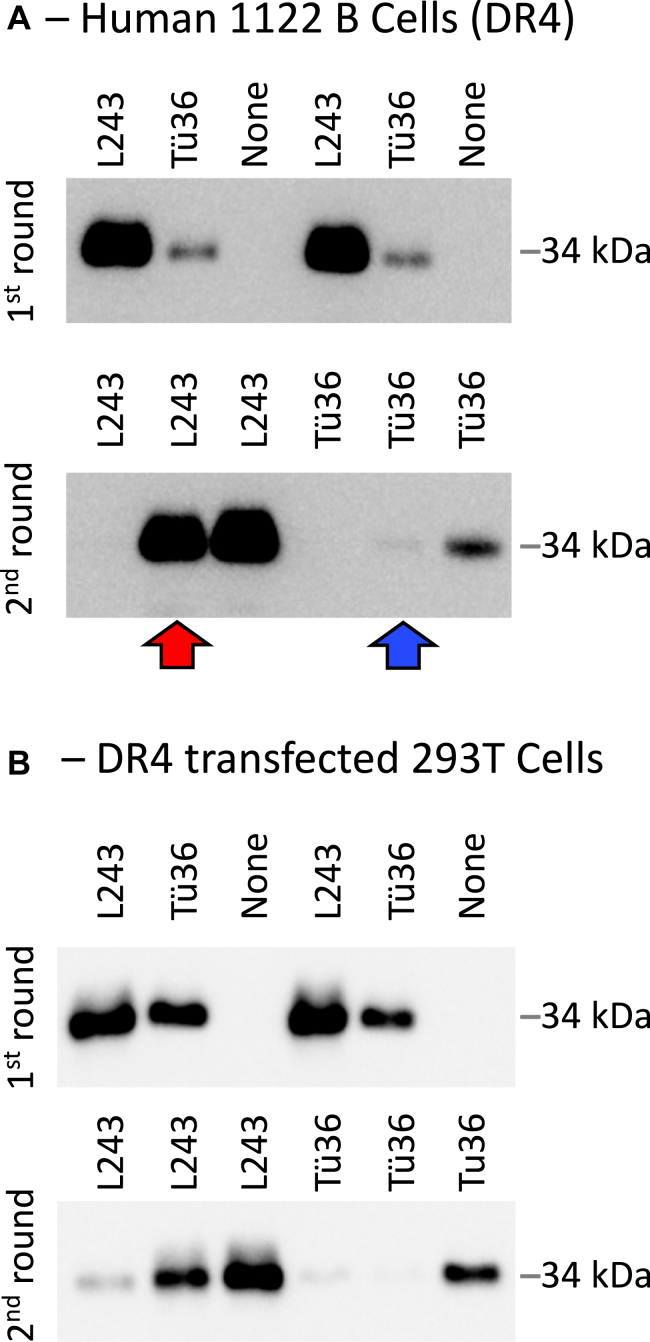
**Tü36 recognizes a subset of HLA-DR4 molecules.** WCL from the indicated cells was prepared as in Figure 1 and subject to two rounds of sequential IP with the indicated mAbs. SN from the first round of IP was used as input for round two. IPs were analyzed for DRA by Western blot as in Figure 1. Shown are representative results from one of two (transfected 293T) to three (1122 B cells) independent experiments. *Gray bar* to the *right* of each blot indicates the position of the 34 kDa molecular weight standard. See the text for an explanation of *red* and *blue arrows*. Densitometry analysis of blots from 1122 B cell samples reveals that Tü36 recognizes 6.7% of class II recognized by the pan-reactive L243 mAb (4%, 5%, and 11% across three independent experiments). IP, immunoprecipitations; WCL, whole cell lysate.

For mouse I-A^k^ class II, the M1 conformer-specific 11-5.2 mAb only immunoprecipitates a subset of I-A^k^ molecules (*i.e.*, M1 paired I-A^k^) (9). To determine if the Tü36 anti-DR mAb exhibits a similar property, the L243 and Tü36 mAb were used in a sequential IP assay, using WCL from DR4-expressing 1122 human B cells (Fig. 3*A*). The results reveal that Tü36 mAb only binds a fraction of total DR4 molecules (5–10%, see figure legend), leaving behind a considerable number of DR molecules that can be recognized by the pan-reactive L243 mAb (Fig. 3*A*, red arrow). This low level of Tü36 reactivity is not due to the use of an insufficient amount of antibody in the first IP, as a second IP with the Tü36 mAb brings down essentially no additional DR molecules (Fig. 3*A*, blue arrow). A similar pattern of reactivity was observed using WCL from 293T cells expressing transfected DR4 (Fig. 3*B*). Thus, similar to the 11-5.2 anti-I-A^k^ mAb, the Tü36 anti-HLA-DR mAb only recognizes a subset of HLA-DR molecules (*i.e.*, M1 paired DR molecules, Fig. 2).

**Figure 4:**
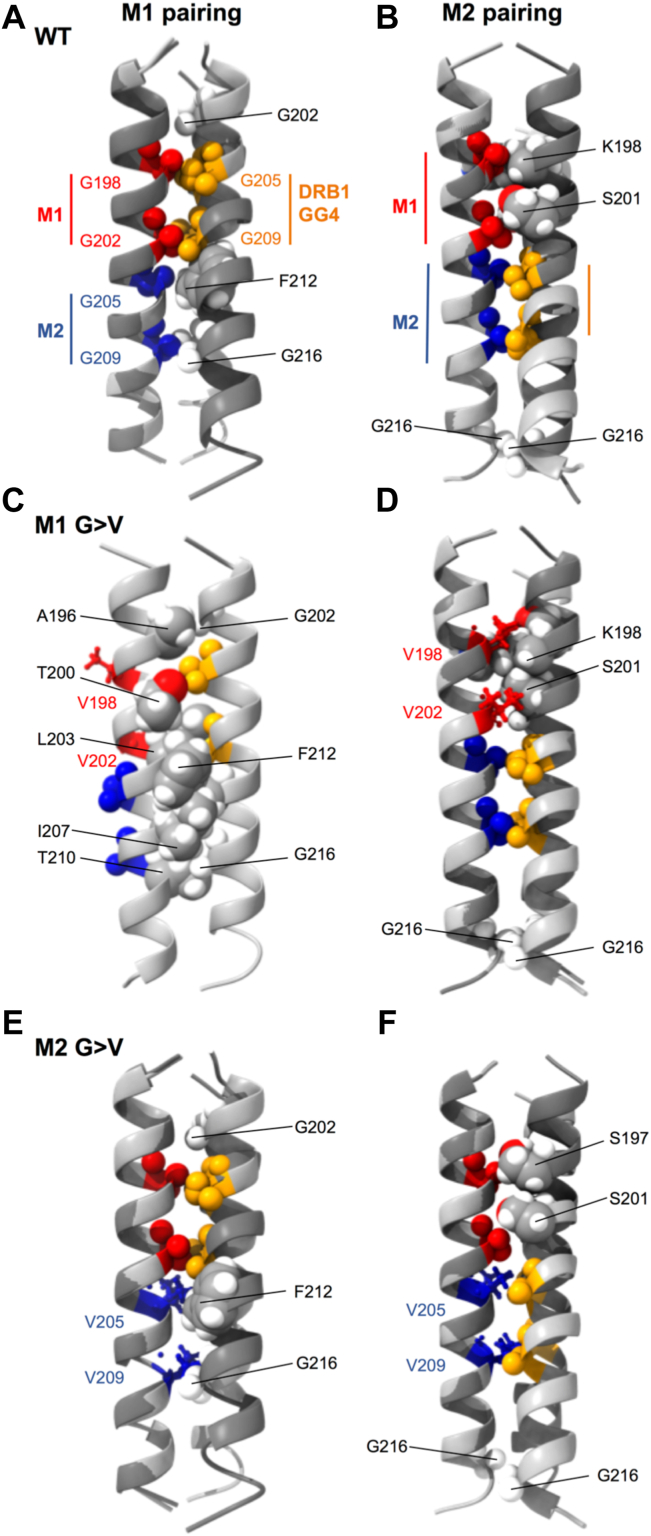
**Modeling of DR transmembrane domain interactions.** For each sequence (apart from panel *C*), the highest ranked (*light gray*) and second-highest ranked (*dark gray*) models obtained from PREDDIMER are overlaid. The two GxxxG motifs in the DRA TM domain are shown as *red spheres* (M1) and *blue spheres* (M2), and the GxxxG motif in DRB1 is shown in *orange*. Additional residues packed at the helix-helix interface are shown as *spheres*, colored by the element, and labeled. Residues that form part of a G>V mutation are shown in *ball* and *stick representation*. *A* and *B*, wild-type sequences yield heterodimers stabilized by packing of either the M1 motif (panel *A*) or the M2 motif (panel *B*) against the single DRB1 motif. *C*, mutation of the M1 motif to valine (G198V, G202V) prevents M1 pairing of the TM domains and more broadly prevents packing of the Gly-rich faces of the two TM domains in all structures obtained. The top-ranked model is shown here (additional models detailed in Fig. S1) in which the DRB1 GxxxG motif packs against bulky and/or polar residues in DRA while the M1/M2 motifs are exposed to lipids. *D*, M2-pairing of the helices is much more tolerant to mutation, yielding models similar to wild-type and further stabilized by close packing of downstream G216 in DRA with G216 in DRB1. *E* and *F*, mutation of the M2 motif to valine (G205V, G209V) yields heterodimers very similar to wild-type, with the top-ranked models illustrating both M1-paired (panel *E*) and M2-paired (panel *F*) heterodimers.

The role of GxxxG motifs in MHC class II TM domain pairing was originally investigated in WT HLA-DR molecules and included *in silico* modeling of TM domain interactions using the CNS searching of helix interactions (CHI) platform (5). The idea of GxxxG motif pairing and *in silico* modeling with CHI was then extended to mouse I-A^k^ class II and the impact of M1 and M2 G>V mutations was also investigated (10). In this report, *in silico* modeling of the impact of M1 and M2 G>V substitutions on TM domain pairing is extended to HLA-DR using the more recently developed PREDDIMER web tool (20) (Fig. 4). PREDDIMER produces a set of structures ranked by the goodness-of-fit for surface complementarity, and all returned structures are summarized in Table S1 of Supporting Information. Figure 4 shows the two highest-ranked structures for each DRA/DRB1 TM domain heterodimer investigated. The WT DRA/DRB1 TM heterodimers are well-packed on the glycine-rich faces of both proteins and exhibit both M1- and M2-based TM domain pairing (Fig. 4, Panels *A* and *B*). These structures are right-handed helical dimers further reflecting the interaction of GxxxG motifs. Extension of the analysis to a DRA M1 G>V sequence (Fig. 2*A*) paired with WT DRB1 results in complete disruption of M1-based TM domain pairing and more broadly prevents normal packing of TM glycine residues (Figs. 4*C* and S1) but does yield well-packed models with M2-based TM domain pairing (Fig. 4*D*). Finally, extension of the analysis to a DRA M2 G>V sequence (Fig. 2*A*) paired with WT DRB1 results in structures very similar to those observed in the WT; one set of structures exhibits M1-based pairing (Fig. 4*E*) and the second set exhibits an M2-like packing between the introduced M2 valine residues (V205 and V209) and the DRB1 GxxxG motif (Fig. 4*F*). For comparison, results of PREDDIMER modeling of I-A^k^ TM domain interactions are shown in Fig. S2. Overall, PREDDIMER modeling is consistent with the Tü36 mAb binding results (Figs. 2 and 7 below). WT DRA and DRB can exhibit either M1 or M2-based TM domain pairing, with the M1-paired form supporting Tü36 mAb binding. The introduction of a DRA M1 G>V substitution prevents M1-based pairing (but allows M2-based pairing), which results in DR molecules impaired for Tü36 binding. In contrast, the introduction of a DRA M2 G>V substitution is well-tolerated and allows both M1-based pairing and an M2-like form of TM domain association, with the ability for M1-pairing allowing for wild-type levels of Tü36 binding. In further studies detailed below, this *in silico* analysis is extended to the entire HLA-DR molecule.

**Figure 5:**
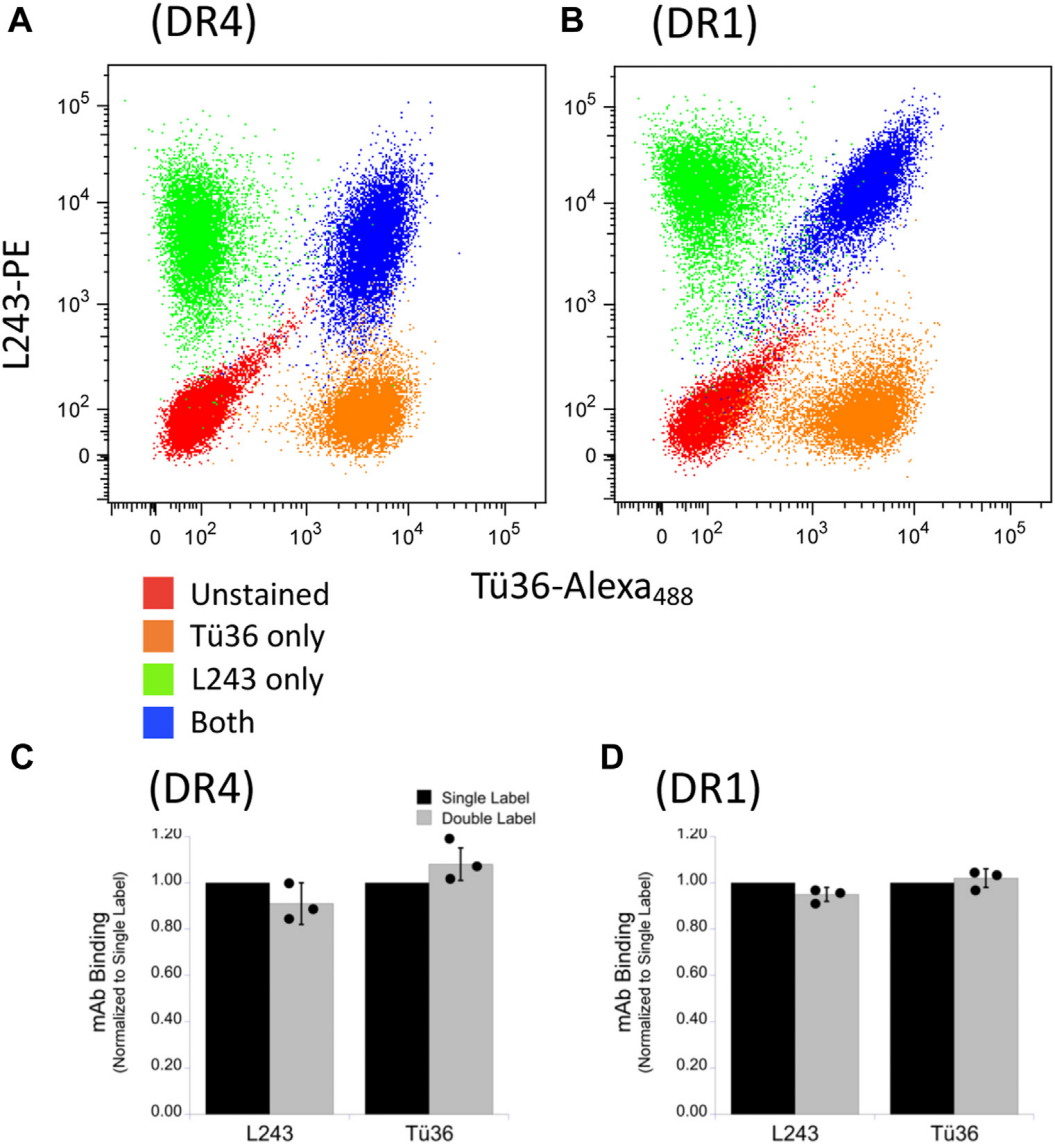
**Simultaneous binding of L243 and Tü36 anti-DR monoclonal antibodies.***A* and *B*, human B cells expressing either DR4 or DR1 were stained concurrently with L243-PE and/or Tü36-Alexa_488_ and analyzed by flow cytometry. *C* and *D*, the level of mAb binding across multiple experiments is compared. The binding of each mAb in isolation was normalized to a value of 1.0 and the relative binding of that mAb in the presence of the partner was determined. Bars indicate ±1 SD from across three independent experiments.

In the murine I-A^k^ system, the 10-3.6 and 11-5.2 mAbs (specific for total and M1-paired I-A^k^ class II respectively) are able to bind to cells/class II molecules at the same time (9, 11). This means that the footprints of the 10-3.6 and 11-5.2 mAbs on the I-A^k^ class II molecule do not overlap (*i.e.*, they bind distinct non-overlapping epitopes). This is a convenient property as cells can be simultaneously stained with both antibodies as a readout for total (10-3.6 binding) and M1 paired (11-5.2 binding) I-A^k^ class II expression. To determine if the same relationship holds for the Tü36/L243 pair of anti-DR mAbs, human B cells expressing either HLA-DR4 or HLA-DR1 were stained with Tü36 and L243 both singly and in combination (Fig. 5). In both cases, the binding of one mAb does not significantly change the binding of the other. To further probe any interactions between the two anti-DR mAb, the impact of pre-binding of a 100-fold molar excess of unlabeled anti-DR mAb on the subsequent binding of fluorochrome-labeled anti-DR mAb was determined (Fig. S3). Under these conditions, binding of unlabeled anti-DR mAb blocks binding of its fluorochrome-labeled version by >99% and inhibits binding of the alternative anti-DR mAb by ∼50%. Here, it should be noted that pre-binding of unlabeled anti-DR mAb may block the binding of a second anti-DR mAb to a different epitope by cross-linking/clustering DR molecules and decreasing epitope accessibility without actually binding the other mAb’s epitope. Taken in total, the results presented in Figure 5 and Fig. S3 demonstrate limited direct competition between the two mAbs for DR binding, suggesting they bind minimally- or non-overlapping epitopes. This means that cells can be stained *simultaneously* with both mAb as a concurrent readout of total and M1 paired HLA-DR expression.

The results presented thus far reveal that while the L243 anti-DR mAb binds all DR class II molecules, the Tü36 anti-DR mAb only binds a subset of DR molecules, the formation of which requires the presence of the DRA TM domain M1 GxxxG dimerization motif (*i.e.*, M1-paired HLA DR) (5, 10). Below, the fine specificity of the Tü36 mAb is further defined.

### Tü36 mAb HLA-DR allele reactivity

Having established the selective binding of Tü36 to M1 paired HLA-DR4 molecules, the analysis of Tü36 reactivity was expanded using a Single Antigen Bead (SAB) assay to determine the reagent’s allele reactivity pattern. The purpose of this analysis was two-fold: First, the analysis will define the range of HLA class II alleles that can be studied with the Tü36 antibody. Second, it may provide additional insight into the epitope recognized by Tü36. For the SAB assay, various HLA-DR alleles are conjugated to an array of microsphere beads of different inherent fluorescence. Beads are probed with an anti-HLA antibody, followed by a fluorochrome-labeled secondary reagent and the multiplex bead sample is then analyzed by Luminex flow cytometry. According to the manufacturer, bead-conjugated HLA molecules are full-length (*i.e.*, they include TM and cytoplasmic domains) and are stabilized via a proprietary approach.

**Figure 6:**
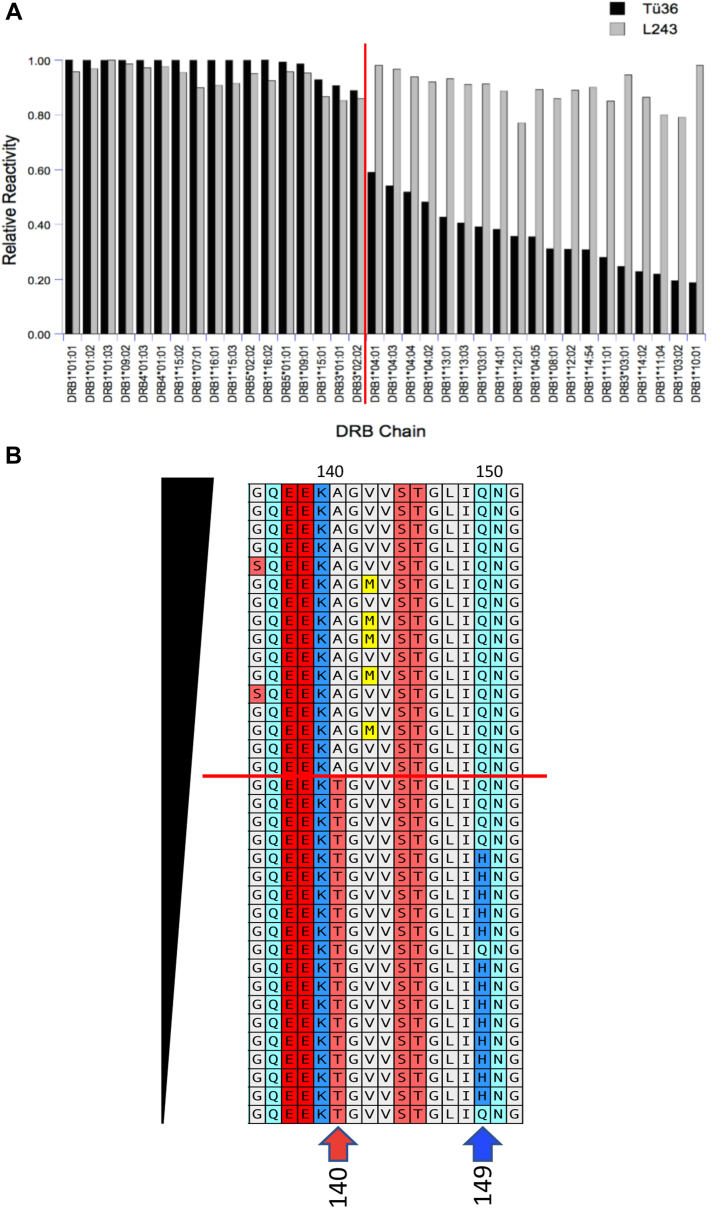
**DR allele reactivity of the Tü36 Anti-DR monoclonal antibody.***A*, the allele reactivity of the L243 and Tü36 mAbs was determined by Single Antigen Bead analysis (see Experimental procedures). For each mAb the MFI of staining of each bead was normalized to the brightest signal for that reagent. The level of staining was ordered from highest to lowest Tü36 binding (*left* to *right*). The *vertical red line* indicates a “break” in Tü36 mAb reactivity. *B*, the amino acid sequence (residues 134–151) of each SAB-interrogated DR allele is ordered based on Tü36 reactivity (highest [*top*] to lowest [*bottom*]—see Fig. S2 for ordered alignment of complete amino acid sequences). The *horizontal red line* indicated the break in Tü36 reactivity (*vertical red line* in panel *A*). The *red* and *blue arrows* indicate amino acid residues that track with Tü36 mAb reactivity (see text).

Results of the SAB analysis are presented in Figure 6*A*, with the various DR alleles ordered from left to right in descending order of Tü36 mAb reactivity. The pan-reactive L243 mAb binds each bead (allele) to a similar level, consistent with the known broad reactivity of this reagent (16) and in line with the manufacturer’s assertion that similar numbers of HLA molecules are conjugated to each bead. The Tü36 mAb exhibits a distinctly different pattern of reactivity. To the left of the vertical red line are DR alleles with similarly high levels of Tü36 reactivity. To the right of the line is a set of DR alleles with progressively decreasing levels of Tü36 reactivity.

**Figure 7:**
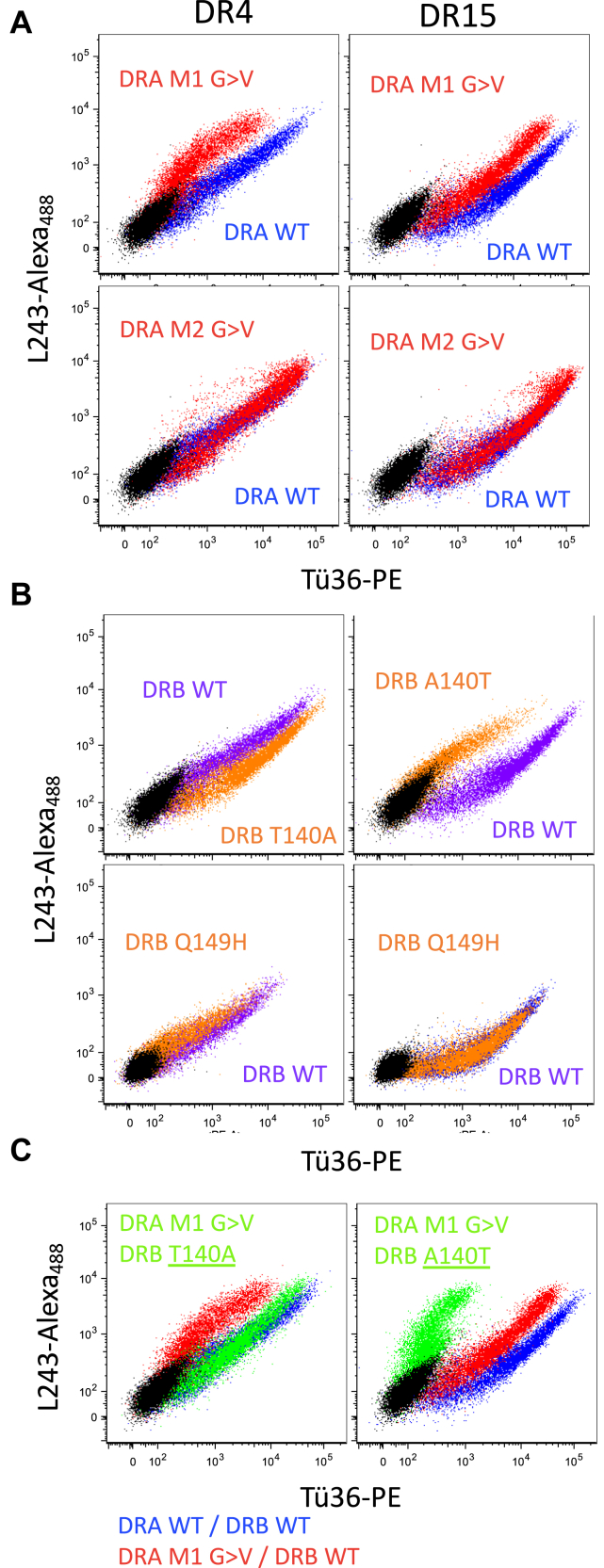
**Double-label analysis of the impact of HLA-DR mutations on Tü36 mAb binding.** 293T cells were transfected with the indicated DRA and DRB chains and then stained with both L243-Alexa_488_ and Tü36-PE. Stained cells were analyzed by flow cytometry. *Black dots* illustrate the staining of non-DR-transfected 293T cells. Shown are representative results from one of three independent experiments. *A*, TM domain GxxxG motif mutants. *B*, DRB 140 and DRB 149 mutants. *C*, combine mutants.

To determine if the DRA TM domain M1 GxxxG motif is important for Tü36 mAb binding to DR alleles other than DR4 (Fig. 2), the level of L243 and Tü36 mAb binding to 293T cells expressing either DR4 or DR15 (a high reactivity allele, Fig. 6*A*) was determined (Fig. 7*A*). In both cases, Tü36 and L243 robustly bind cells expressing WT DR molecules with higher relative Tü36 binding seen for DR15, consistent with the higher level of Tü36 DR15 reactivity seen in the SAB assay (Fig. 6*A*). In both cases, the binding of Tü36 is selectively impaired by a DRA M1 GxxxG>VxxxV (M1 G>V) mutation, while an M2 G>V mutation has little impact on Tü36 mAb binding. In fact, there is a small but reproducible increase in Tü36 mAb binding to the M2 G>V mutant (Fig. S4). Thus, the presence of the DRA TM domain M1 GxxxG motif, which is necessary for the formation of M1-paired class II (5, 10, 11), is critical for Tü36 mAb binding to multiple HLA-DR alleles.

To explore the possibility that other DRB allelic amino acid polymorphisms impact Tü36 mAb reactivity (DRA is essentially monomorphic at the amino acid level), the amino acid sequences of SAB-interrogated DRB alleles were aligned in order of decreasing Tü36 mAb reactivity (Figs. 6*B* and S5). While there are multiple areas of DRB polymorphism that track with mAb reactivity (discussed below), residue 140 is particularly interesting. For all DRB alleles with high Tü36 reactivity, there is an alanine in this position, whereas for all alleles with lower Tü36 reactivity, there is a threonine (Fig. 6*B*, red arrow). Also of note, the TM domain of the tested DRB alleles is highly conserved (Fig. S5) indicating that variable Tü36 allele reactivity is *not* due to polymorphisms of the DRB TM domain GxxxG motif, which could impact pairing with the alpha chain M1 or M2 GxxxG motifs.

To directly test the impact of DRB residue 140 on Tü36 mAb recognition, 293T cells were transfected with one of four different DRB chains (along with DRA), and Tü36 mAb reactivity was determined. DRB1∗04:01 (DR4) has a threonine at position 140, so this was changed to alanine (DR4 T140A), whereas DRB1∗15:01 (DR15) has an alanine at position 140 so this was changed to threonine (DR15 A140T). Cells were co-stained with both L243 and Tü36 and analyzed by flow cytometry (Fig. 7*B*). In all four cases, transfected cells bound to detectable levels of both antibodies. However, when a DR4 T140A substitution was tested there was an increase in Tü36 mAb binding (Fig. 7*B*, rightward shift in staining), consistent with alanine being present at this position in high Tü36 binding alleles (Fig. 6). In contrast when a DR15 A140T substitution was tested, there was a decrease in Tü36 mAb binding (Fig. 7*B*, leftward shift of staining, also see Fig. S4) consistent with threonine being present at this position in low Tü36 binding DRB alleles (Fig. 6).

There are three additional regions of DRB polymorphism that track closely with Tü36 mAb reactivity (Fig. S5): residues 10 to 12, residue 149, and residue 233 (residue 233 is in the molecule’s cytoplasmic domain). DRB 149 was of particular interest because like DRB 140, residue 149 is in the membrane-proximal immunoglobulin-like domain of DRB and there is a notable correlation between two alternative amino acid residues (Q or H) and Tü36 mAb binding with glutamine (Q) being present at this position in high/moderate Tü36 reactivity alleles (Fig. 6*B*, blue arrow). The role of DRB 149 in Tü36 binding was investigated by transfecting 293T cells with either WT or Q149H mutants of DRB1∗04:01 or DRB1∗15:01 (along with DRA) and then staining with both L243 and Tü36 (Fig. 7*B*). For the moderate-Tü36 binding DR4 allele there was a modest but reproducible decrease in Tü36 binding with the Q149H mutation (Figs. 7*B* and S4), whereas for the high-Tü36 binding DR15 allele, there was little if any effect of the Q149H mutation on Tü36 mAb binding (Fig. 7*B*). Thus, while both DRB1 140 and 149 residues impact Tü36 mAb binding, the 140 A/T polymorphism has a greater impact than the 149 Q/H polymorphism.

This analysis was extended by pairing a WT or TM domain M1 G>V mutant DRA with a WT or 140 mutant DRB and measuring the extent of Tü36 mAb binding (Fig. 7*C*). In the case of DR4, the DRA M1 G>V mutation on its own decreases Tü36 binding whereas the introduction of an additional DRB T140A mutation increases binding, such that the DR4 M1 G>V/DRB T140A compound mutant exhibits Tü36 binding similar to the WT molecule (Fig. 7*C*). For the DR15 molecule, the DRA M1 G>V mutation and the DRB A140T mutation both decrease Tü36 binding such that the DR15 M1 G>V/DRB A140T compound mutant exhibits profoundly decreased Tü36 binding (Fig. 7*C*). Thus, for both HLA-DR molecules, each region of the class II dimer (*i.e.*, the TM domain and the region around DRB 140) impacts Tü36 mAb binding independently. While the TM domain likely impacts Tü36 mAb binding *via* an allosteric effect, DRB 140 may act *via* an allosteric effect or may be directly involved in mAb binding (see below).

### Molecular modeling of full-length HLA-DR structure

**Figure 8:**
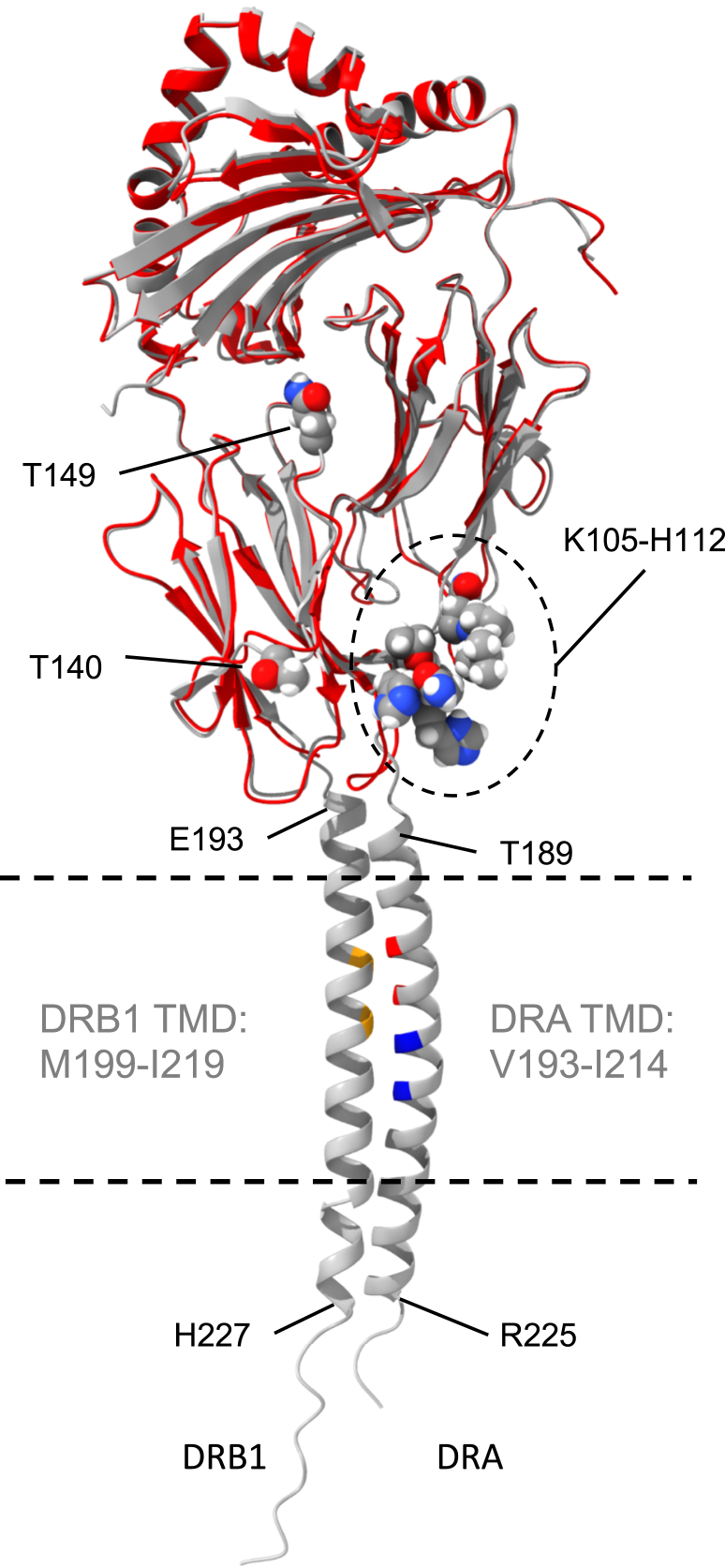
**Overlay of alphaFold2 model of wld-type DR4 and DR4 crystal structure.** The structure of the AlphaFold2 model of full-length DR4 (see text, *gray trace*) and crystal structure of the DR4 extracellular domain (pdb file 7NZE, *red trace*) were aligned using the Matchmaker tool in Chimera X1.4. The alignment yields an RMSD between 179 pruned atom pairs of 0.843 Å. Key residues/regions of interest in the extracellular domain are highlighted as *spheres*. The limits of a long helical domain, containing the TM domain, are also labeled and the TM GxxxG motifs are colored as in Figure 4.

The structure of the full-length HLA-DR molecule (including the TM and cytoplasmic domains) was modeled by submitting the sequences of DRA 01:01 (amino acid residues 1–229) and DRB1 04:01 (residues 1–237) to AlphaFold2 within the ColabFold environment (21). Five structures were returned by AlphaFold2, and the highest ranked of these is shown in Figure 8 (confidence levels of prediction are described in Experimental procedures and shown in Fig. S6). The AlphaFold2 structure of the extracellular domain was very similar to the DR4 crystal structure (PDB 7NZE, shown in red in Fig. 8) and reinforces the close physical proximity of DRB residues T140 and Q149 (which impact Tü36 mAb binding, Fig. 7) and a high flexibility region of the protein (*i.e.*, DRB 105–112 (22), – discussed below). The model then predicts that two short poorly structured regions connect the extracellular domain to two extended helical regions that contain the TM domains followed by two very short unstructured cytoplasmic tails. The length of the helical regions is notable (36 amino acids for DRA and 34 for DRB) as they are longer than is necessary for a conventional TM domain. Indeed, DeepTMHMM (23) predicts TM domain lengths of 21 to 22 residues (annotated in Fig. 8). The TM domains pack in the AlphaFold2 model on their Gly-rich faces, with the helix–helix interface containing the M1 and M2 motifs in DRA and the GxxxG motif in DRB. However, instead of a GAS_right_ interaction of the GxxxG motifs, the AlphaFold2 model predicts an Ala-zipper style coiled-coil interaction between small residues at the *a* and *d* positions of a heptad repeat (6). This packing is characteristic of what is known as an antiparallel GAS_left_ interaction of small residues and a shallow left-handed crossing angle. Inspection of the DRA and DRB1 TM domain sequences (Fig. S6, bottom panel) reveals that in addition to GxxxG motifs, both also contain putative coiled-coil motifs with Gly residues at the *a* and *d* positions. Given that these motifs are statistically the most over-represented helix–helix interaction motifs found in membrane proteins of known structure (6), it is likely that AlphaFold2 has aligned these regions in DRA and DRB1 accordingly and thus predicts a GAS_left_ interaction. However, this mode of interaction is not supported by the mutagenesis data here nor the mutagenesis data in the literature (10, 11) and can thus be ruled out in favor of the GAS_right_ interaction of TM domains.

The non-TM domain ends of the extended helices contain polar amino acids, with positively charged residues enriched in the cytoplasmic portion. This extended helical structure might allow the TM domains to access multiple modes of interaction that require different side-by-side registers (*i.e.*, M1 *versus* M2 pairing, see Fig. 4 PREDDIMER modeling). Because of the rigid nature of these helical domains, any changes in TM domain orientation would change the spacing of the N-terminal ends of the helical regions (*i.e.*, DRA T189 and DRB E193) and would be more readily transmitted to the molecule’s extracellular domain to induce a conformational shift. However, attempts to model conformational variants using AlphaFold2 by introducing M1 and M2 G>V mutations did not yield new structures (data not shown), likely due to the continued alignment of alternative (GAS_left_) motifs that were not impacted by these mutations. More broadly, the program has acknowledged limitations that constrain its ability to predict the structure of protein conformational variants (24, 25). Therefore, while the AlphaFold2 models have provided valuable information, the modeling of conformational variants will require further work.

### M1-paired HLA-DR molecules and lipid rafts

**Figure 9:**
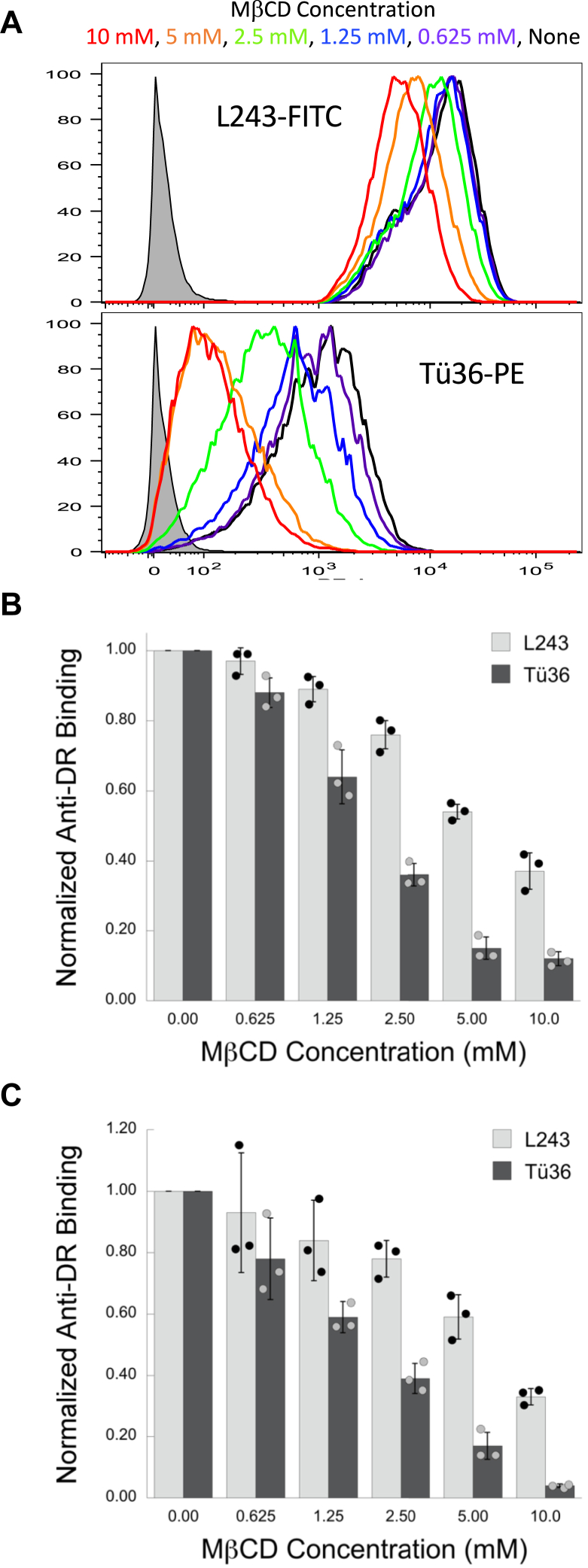
**Impact of MβCD treatment on anti-DR mAb binding.** DR4-expressing 1122 human B cells were treated with the indicated concentration of MβCD and then co-stained with L243-FITC and Tü36-PE. Stained cells were analyzed by flow cytometry. *A*, histograms of mAb staining at each MβCD concentration as indicated. *Filled-in gray histogram* is from unstained cells. *B*, the level of mAb binding to 1122 B cells across three experiments is compared. The binding of each mAb in the absence of MβCD treatment was set to a value of 1.0 and other values normalized to that level of mAb binding. Bars indicate ±1 SD from across three independent experiments. *C*, impact of MβCD treatment on Tü36 and L243 binding to Raji B cells. Results compiled from across three independent experiments as for panel *B*.

Previous studies demonstrate that M1-paired mouse I-A^k^ class II molecules are enriched in cholesterol-rich membrane domains colloquially termed “lipid rafts” (9). Therefore, we sought to investigate the role of lipid rafts in Tü36 HLA-DR mAb binding. For mouse I-A^k^ class II, Roy *et al.* (26) reported that treatment of macrophages with 10 mM methy-β-cyclodextrin (MβCD), which will extract membrane cholesterol and disrupt lipid rafts, results in a selective decrease in 11-5.2 mAb binding. For human HLA-DR, Kropshofer *et al.* have shown that treatment of DR4-expressing WT-51 human B cells with 5 mM MβCD results in a selective decrease in Tü36 mAb binding [compared to L243 binding (27)]. To extend these findings, we treated DR4-expressing 1122 human B cells with a range of MβCD doses and determined the impact on binding of the L243 and Tü36 anti-DR mAbs (Fig. 9, *A* and *B*). With increasing doses of MβCD, there is a more precipitous decrease in Tü36 mAb binding compared to L243. The same trend is seen if Raji cells (which co-express DR3 [DRA/DRB1∗03:01] and DR10 [DRA/DRB1∗10:01]) are treated with MβCD (Fig. 9*C*). These results are consistent with the findings of Roy and Krosphofer and reveal that cholesterol-rich lipid rafts are central to the immunobiology of M1-paired HLA-DR molecules recognized by the Tü36 mAb.

## Discussion

Results presented in this report extend the paradigm of a differential pairing of TM domain M1 *versus* M2 GxxxG motifs from mouse to human MHC class II molecules by identifying Tü36 as a mAb that selectively recognizes M1-paired HLA-DR molecules. The results also define the allele reactivity of Tü36 and identify amino acid residues outside of the DR molecule’s TM domain that impact Tü36 mAb binding. *In silico* modeling provides additional insight into the impact of differential TM domain pairing on the overall structure of the HLA-DR molecule. These findings add another layer of complexity and sophistication to the immunobiology of human MHC class II molecules.

Inceptive biochemical and computational studies identified multiple GxxxG dimerization motifs in the TM domains of MHC class II molecules across many species and laid open the possibility of multiple modes of TM domain pairing (5). Subsequent studies of the murine I-A^k^ class II molecule established two forms of TM domain association (*i.e.*, M1-and M2-pairing) and revealed the impact of TM domain pairing on the expression of the Ia.2 epitope recognized by the 11-5.2 mAb (10, 11). Additional studies using the 11-5.2 mAb, which specifically binds M1-paired I-A^k^ class II, demonstrate selective partitioning of M1-paired I-A^k^ molecules into membrane microdomains colloquially termed “lipid rafts” (9) and reveal the unique signaling properties of these raft-resident class II molecules (13, 14). 11-5.2 mAb blocking studies reveal the central role of M1 paired I-A^k^ molecules in *in vivo* T cell activation (12) as well as *in vitro* B cell–T cell interactions (9). Studies of B cell receptor (BCR)-mediated antigen processing revealed the selective association of M1-paired I-A^k^ class II with endosomal antigen-BCR complexes in a putative MHC class II peptide loading complex (15) and demonstrated selective loading of peptides derived from BCR-bound cognate antigen onto M1-paired class II molecules (1, 15). Finally, unpublished studies of M1 and M2-paired I-A^k^ have revealed only about a 50% overlap in the pools of antigenic peptides (*i.e.*, peptidomes) bound to each conformer (J. Drake – unpublished). These prior studies in combination with the results presented in this report suggest a similar level of sophistication for human HLA-DR molecules. Computational modeling of human HLA-DR revealed putative M1-and M2 paired TM domain conformations (Fig. 4), both stabilized by interactions between GxxxG motifs, and these models are in full agreement with all experimental findings. AlphaFold2 structures of full-length HLA-DR predict extended TM domain-containing helical regions that could readily transmit changes in TM domain orientation to changes in the extracellular domain, thus impacting mAb binding (Fig. 8).

One outstanding question related to this story is the exact region of the DR molecule bound by the Tü36 M1 conformer-specific mAb (*i.e.*, the Tü36 epitope). While a complete answer to this question will require additional work, we can draw a few meaningful conclusions from what is currently known. First, the Tü36 mAb can immunoprecipitate DR molecules from cell lysates produced under relatively harsh conditions (*i.e.*, RIPA buffer). This suggests that the Tü36 epitope does not require any “accessory proteins” that are loosely associated with the DR molecule (*e.g.*, a tetraspan protein (28, 29)). Second, since the L243 and Tü36 mAbs can simultaneously bind DR molecules they must bind minimally/non-overlapping epitopes. The L243 epitope has been partially mapped and includes DRA residues 18 and 39, which are located in the membrane-distal region of the molecule near the peptide binding groove (16). This suggests that Tü36 may bind a more membrane-proximal region of the DR molecule. Finally, the impact of a DRB 140 A>T substitution of Tü36 mAb binding is partial (*i.e.*, mutation of this residue does not completely abolish Tü36 mAb binding). Thus, it is likely that the residue is indirectly involved in mAb binding. It may be at/near the perimeter of the Tü36 epitope or, alternatively, it may impact mAb binding via an allosteric effect.

Interestingly, DRB 140 is near a region of the protein reported to be highly flexible (*i.e.*, DRB 105–112 (30), Fig. 8). This region is also predicted by AlphaFold2 to be highly flexible, as indicated by the low pLDDT confidence measure in the B-factor field. This is most easily seen in Fig. S6, where DRB residues 105 to 112 are colored orange (circled in figure), reflecting the low confidence of prediction for this region compared with the surrounding regions. Taken together, it would suggest that this flexible region of the molecule may adopt different conformations depending on the mode of TM domain interaction, thus impacting mAb binding. DRB 140 is also near the CD4 binding site (31), raising the possibility that M1- *versus* M2-paired DR molecules may interact with CD4 in different ways. In any case, additional work is needed to more precisely define the Tü36 mAb epitope and the results of these studies will also yield greater insight into the impact of TM domain pairing on overall HLA-DR structure and function.

The impact of the DRB Q149H mutation on Tü36 mAb binding is subtle but reproducible. Analysis of DR crystal structures in the NCBI structure database with the Protein Data Bank in Europe’s Proteins, Interfaces, Structures and Assemblies (PISA) server (https://www.ebi.ac.uk/pdbe/pisa/) reveals a differential role of these alternative residues in the interaction of the DRB1 chain with DRA (Table 1). In DR molecules possessing glutamine (Q) at DRB 149, there are two hydrogen bonds between this residue and two asparagine residues in DRA. In contrast, DR molecules possessing a histidine (H) at DRB 149 form two hydrogen bonds and two salt bridges between this residue and asparagine residues in DRA. It is tempting to speculate that the additional salt bridges may impact the ability of the molecule to adopt a conformation bearing the Tü36 epitope irrespective of TM domain pairing. This idea will require further investigation.

**Table 1:** HLA-DR inter-chain interactions involving DRB residue 149^a^

DRB allele/mmdb file	DRB residue 149	DRB residue 149 interactions (with DRA chain residues)
DR1/1AQD	Q	H-bond with N 27 H-bond with N 29^b^
DR4/2SEB	Q	H-bond with N 27 H-bond with N 29
DR3/1A6A	H	H-bond with N 27 H-bond with N 29 Salt bridge (two) with N 29
DR11/6CPN	H	H-bond (two) with N 29 Salt bridge (two) with N 29

a mmdb files analyzed at https://www.ebi.ac.uk/msd-srv/prot_int/cgi-bin/piserver.

b AlphaFold2 analysis of DR structure also indicates a hydrogen bond between DRB Q149 and DRA R146.

Finally, to determine if there is a phylogenetic relationship between Tü36 mAb binding, DRB1 amino acids residues 140 and 149, and the various DRB1 allele groups, we developed an amino acid sequence-based phylogenetic tree of the DRB1 alleles tested in the SAB assay (Fig. S7). For this analysis, we used amino acid (as opposed to nucleotide) sequences since we are looking at protein-based characteristics. We also excluded the protein products of non-DRB1 genes such as DRB5. Working from left to right on the resulting phylogenetic tree, we encounter an initial bifurcation that divides all of the alleles that have an alanine at position 140 (*e.g.*, DRB1∗01:01, upper branch) from the alleles that have a threonine at that position (*e.g.*, DRB1∗04:01, lower branch). Interestingly, all of the alleles in the upper branch also have glutamine at position 149 and uniformly exhibit a high level of Tü36 mAb binding (Fig. 6). Shifting the focus to the lower branch, we next encounter a bifurcation that separates the DRB1∗04 allele family from all of the other members of this branch. The DR1∗04 family generally exhibits an intermediate level of Tü36 mAb binding (Fig. 6), has shifted to threonine at position 140 but retains glutamine at position 149. Finally, for the non-DRB1∗04 arm of the lower branch, all of the alleles have a threonine at position 140 and have shifted to histidine at position 149 (save DRB1∗10:01). These alleles uniformly fall on the low end of the Tü36-reactivity spectrum (Fig. 6). Taken together, these results indicate that Tü36 mAb recognition along with the amino acids present at DRB residues 140 and 149 divide DRB1 alleles into three groups. It is tempting to speculate that these three groups have different structural properties (leading to differential Tü36 binding) that may impact their immunological function. This idea awaits further investigation.

In conclusion, the results presented in this report establish the existence of M1 and M2-paired HLA-DR class II conformers by defining a mAb (Tü36) that exhibits selective reactivity toward lipid raft-resident M1-paired HLA-DR molecules. These results build upon previous studies in human (5) and murine (9, 10) class II molecules and are consistent with reports on the structural plasticity of human class II molecules (22, 30, 32, 33). Future work will more precisely define the Tü36 mAb epitope and the fine structural differences between M1-and M2-paired HLA-DR molecules. Incorporation of the idea of HLA-DR conformers into clinical assays such as the screening of potential transplant recipients for anti-HLA alloantibodies or post-transplant for donor-specific antibodies, or in the design and use of immunotherapeutic anti-DR antibodies (https://clinicaltrials.gov/ct2/show/NCT01728207), will likely yield further insight into the immunobiology of these interesting molecules.

## Experimental procedures

### Cell lines

The IHW01122 (1122) and IHW01124 (1124) human B cells were obtained from the Research Cell Bank at the Fred Hutchinson Cancer Research Center and grown in RPMI 1640 media containing 15% FBS and 1 mM sodium pyruvate. Raji cells were grown in the same media as 1122 and 1124 cells. 293T cells were grown as previously reported (11). DR51-expressing K46μ mouse B cells were generated by transfecting K46μ cells (10) with DRA∗01:02 cDNA in pcDNA3.1(−) and DRB5∗01:01 cDNA in pcDNA3.1(−) by electroporation and growth in RPMI 1640 media 10% FBS, 50 μM 2-ME, 1 mM Na-pyruvate, 1× non-essential amino acids (Corning 25-025I) containing 500 μg/ml G418.

### Antibodies

Tü36 (BioLegend 361602), Tü36-FITC (BioLegend 361604), Tü36-PE (BioLegend 361606), L243 (BioLegend 307602), L243-FITC (BioLegend 307632), L243-PE (BioLegend 307606), 423L-btn (Thermo Fisher Scientific MA1-12180), Rabbit anti-DRA (ThermoFisher Scientific PA5-22279), goat anti-rabbit IgG-HRP (Calbiochem 401353).

### 293T cell transfection

293T cells were transfected with the indicated cDNAs in pcDNA3.1+ using Fu-GENE transfection reagent as previously reported (10). After 24 to 48 h, cells were collected, stained, and analyzed by flow cytometry.

### Immunoprecipitation

Cells were lysed in RIPA buffer (50 mM Tris pH 7.5, 150 mM NaCl, 5 mM EDTA, 0.5% deoxycholate 1% NP-40 substitute) containing protease inhibitors (Thermo Fisher Scientific A32955). Lysates were cleared by centrifugation at 16,000*g*. Class II was immunoprecipitated overnight at 4 °C (constant inversion) with 5 to 10 μg of indicated antibody and 10 μl of Protein A-agarose beads (Thermo Fisher Scientific 20334).

### Western blot

Samples were boiled in 1× SDS-PAGE sample buffer and fractionated on a 10% SDS-PAGE gel. The material was transferred to 0.2 μm nitrocellulose (BioRad 1620112) by semi-dry blotting. Blots were blocked with Blotto (5% non-fat dry milk and 0.1% Triton X-100 in borate buffer pH 8.0) containing 10% horse serum. Blocked blots were probed with rabbit anti-DRA (Invitrogen PA5-104395, 1:10,000) followed by goat anti-rabbit IgG-HRP (Calbiochem 401353, 1:25,000), both diluted in Blotto 10% horse serum and then developed with SuperSignal West Dura Extended Duration Substrate (Thermo Fisher Scientific 34076).

### Site-directed mutagenesis

Mutagenesis was done with the Agilent QuikChange mutagenesis kit as previously reported (10). All mutants were confirmed by DNA sequencing before further analysis.

### Flow cytometry

Cells were stained with the indicated antibodies (unlabeled and FITC-labeled antibodies at 1:100 dilution and PE-labeled antibodies at 1:200), washed, and analyzed on a BD FACSymphony A3 running FACSDiva 8.0, collecting 20,000 to 30,000 live cell events/sample. Data were analyzed with FlowJo (V9.9.4).

### Single antigen bead assay

Anti-DR mAb specificity was determined using LabScreen Single Antigen HLA Class II – Group 1 (One Lambda/Thermo Fisher Scientific - LS2AEX01) microsphere beads according to the manufacturer’s protocol except that a phycoerythrin-labeled goat anti-mouse IgG (Southern Biotech 1031-09) was used as the secondary antibody for detection of mAb binding. Microspheres were analyzed on a Luminex 100/200 System (Luminex Corp) instrument with xPONENT software (Luminex Corp) for data acquisition and then HLA Fusion Software (One Lambda/Thermo Fisher Scientific) for analysis.

### Phylogenetic tree assembly

The single-letter amino acid sequences of the indicated DRB1 alleles (https://www.ebi.ac.uk/ipd/imgt/hla/) were entered into http://www.phylogeny.fr/simple_phylogeny.cgi in the “One Click” mode (34).

### Molecular modeling

Computational analyses of DRA∗01:01 and DRB1∗04:01 TM domain heterodimers were performed using the PREDDIMER prediction tool (See Table S1 in Supporting Information for sequences), a surface-based algorithm for prediction of dimer conformations (20). PREDDIMER uses the molecular hydrophobicity potential (MHP) approach to map hydrophobic and hydrophilic properties onto helical surfaces and determine complementarity. The output is a set of structures ranked by quality of packing (FSCOR). The values for FSCOR and crossing angle for all heterodimer models output from PREDDIMER are given in Tables S1-I and S1-II in Supporting Information, along with the details of the analyzed sequences. The full-length wild-type HLA-DR heterodimer, composed of DRA∗01:01 (residues 1–229) and DRB1∗04:01 (residues 1–237), was submitted to AlphaFold2 within the ColabFold environment (21) and the resulting structure colored according to the pLDDT confidence measure in the B-factor field. The highest confidence regions are colored darkest blue, the lowest confidence regions are colored orange, and the intermediate confidence regions are yellow-light blue. All molecular graphics and analyses from both PREDDIMER and AlphaFold2 were produced using UCSF Chimera X, developed by the Resource for Biocomputing, Visualization, and Informatics at the University of California, San Francisco, with support from NIH P41-GM103311 (35). The transmembrane domains were predicted using DeepTMHMM (https://www.biorxiv.org/-https://doi.org/10.1101/2022.04.08.487609).

### MβCD treatment of B cells

1122 or Raji B cells in HBSS 0.1% BSA were treated with the indicated concentration of methyl-β-cyclodextrin (MβCD, Sigma cat. # C4555) for 20 min at 37 °C. Cells were then chilled and stained with fluorochrome-labeled anti-DR mAbs for 20 min on ice. Samples were washed once and then analyzed by flow cytometry (unfixed).

## Data availability

All data is included in this document.

## Supporting information

This article contains supporting information.

## Conflict of interest

The authors declare that they have no conflicts of interest with the contents of this article.
