# Supplementary material for: Differential pairing of transmembrane domain GxxxG dimerization motifs defines two HLA-DR MHC class II conformers
Source: J Biol Chem. 2023 May 27;299(7):104869. doi: 10.1016/j.jbc.2023.104869 (PMC10320510; doi:10.1016/j.jbc.2023.104869)
Supplement: Supporting information [file mmc1.docx]

**Supporting Information** - Differential pairing of transmembrane domain GxxxG dimerization motifs defines two HLA-DR MHC class II conformers – Drake et al.

**Table SI-I – PREDDIMER parameters for all predicted HLA-DR TMD models^1^**

| rank | Fscor | DRA 01:01 | DRB1 04:01 | crossing angle | RH / LH |
| --- | --- | --- | --- | --- | --- |
| 1 | 3.333 | WT res. 191-216 | WT res. 198-223 | -0.2 | RH |
| 2 | 2.879 | WT res. 191-216 | WT res. 198-223 | -40.1 | RH |
| 3 | 2.547 | WT res. 191-216 | WT res. 198-223 | 30.1 | LH |
| 4 | 2.371 | WT res. 191-216 | WT res. 198-223 | 55 | LH |
|  |  |  |  |  |  |
| 1 | 2.687 | M1 G>V res. 191-216 | WT res. 198-223 | 5.1 | LH |
| 2 | 2.637 | M1 G>V res. 191-216 | WT res. 198-223 | 35.1 | LH |
| 3 | 2.454 | M1 G>V res. 191-216 | WT res. 198-223 | 60.1 | LH |
| 4 | 2.375 | M1 G>V res. 191-216 | WT res. 198-223 | -45 | RH |
| 5 | 1.751 | M1 G>V res. 191-216 | WT res. 198-223 | -20 | RH |
| 6 | 1.74 | M1 G>V res. 191-216 | WT res. 198-223 | 50 | LH |
| 7 | 1.7 | M1 G>V res. 191-216 | WT res. 198-223 | -55 | RH |
| 8 | 1.478 | M1 G>V res. 191-216 | WT res. 198-223 | -60 | RH |
|  |  |  |  |  |  |
| 1 | 3.141 | M2 G>V res. 191-216 | WT res. 198-223 | 5.8 | LH |
| 2 | 2.94 | M2 G>V res. 191-216 | WT res. 198-223 | -25.2 | RH |
| 3 | 2.761 | M2 G>V res. 191-216 | WT res. 198-223 | -55 | RH |
| 4 | 2.358 | M2 G>V res. 191-216 | WT res. 198-223 | 50 | LH |
| 5 | 1.922 | M2 G>V res. 191-216 | WT res. 198-223 | 20.3 | LH |
|  |  |  |  |  |  |
| 1 | 3.681 | WT res. 191-216 | WT res. 191-218 | -50 | RH |
| 2 | 3.568 | WT res. 191-216 | WT res. 191-218 | -5.1 | RH |
| 3 | 3.118 | WT res. 191-216 | WT res. 191-218 | 35 | LH |
| 4 | 3.068 | WT res. 191-216 | WT res. 191-218 | 60 | LH |
| 5 | 2.671 | WT res. 191-216 | WT res. 191-218 | 20.4 | LH |
|  |  |  |  |  |  |
| 1 | 3.055 | M1 G>V res. 191-216 | WT res. 191-218 | -20 | RH |
| 2 | 2.951 | M1 G>V res. 191-216 | WT res. 191-218 | 4.8 | LH |
| 3 | 2.646 | M1 G>V res. 191-216 | WT res. 191-218 | -55.2 | RH |
| 4 | 1.962 | M1 G>V res. 191-216 | WT res. 191-218 | 50 | LH |
| 5 | 1.49 | M1 G>V res. 191-216 | WT res. 191-218 | 20.2 | LH |
|  |  |  |  |  |  |
| 1 | 2.96 | M2 G>V res. 191-216 | WT res. 191-218 | -30.1 | RH |
| 2 | 2.877 | M2 G>V res. 191-216 | WT res. 191-218 | 10.1 | LH |
| 3 | 2.763 | M2 G>V res. 191-216 | WT res. 191-218 | 40 | LH |
| 4 | 2.404 | M2 G>V res. 191-216 | WT res. 191-218 | 60 | LH |
| 5 | 2.098 | M2 G>V res. 191-216 | WT res. 191-218 | -10.2 | RH |
| 6 | 1.687 | M2 G>V res. 191-216 | WT res. 191-218 | -50.2 | RH |

1 Included are the Fscor, a reflection of the goodness of fit for each model, the sequences submitted for analysis, the crossing angle, and the handedness of each model (RH: right handed, LH: left-handed).

**Table SI-II – PREDDIMER parameters for all predicted I-A^k^ TMD models**

| rank | Fscor | AaK | AbK | crossing angle | RH / LH |
| --- | --- | --- | --- | --- | --- |
| 1 | 3.675 | WT res. 195-220 | WT res. 197-222 | -5.1 | RH |
| 2 | 2.814 | WT res. 195-220 | WT res. 197-222 | -60 | RH |
| 3 | 2.794 | WT res. 195-220 | WT res. 197-222 | 30.1 | LH |
| 4 | 2.580 | WT res. 195-220 | WT res. 197-222 | -30.2 | RH |
| 5 | 2.490 | WT res. 195-220 | WT res. 197-222 | 60 | LH |
|  |  |  |  |  |  |
| 1 | 3.079 | M1 G>V res. 195-220 | WT res. 197-222 | -15.1 | RH |
| 2 | 2.915 | M1 G>V res. 195-220 | WT res. 197-222 | -10.1 | RH |
| 3 | 2.713 | M1 G>V res. 195-220 | WT res. 197-222 | -40.1 | RH |
| 4 | 2.553 | M1 G>V res. 195-220 | WT res. 197-222 | -55 | RH |
| 5 | 2.503 | M1 G>V res. 195-220 | WT res. 197-222 | 40 | LH |
| 6 | 2.306 | M1 G>V res. 195-220 | WT res. 197-222 | 15.3 | LH |
| 7 | 1.568 | M1 G>V res. 195-220 | WT res. 197-222 | 10.5 | LH |
|  |  |  |  |  |  |
| 1 | 3.281 | M2 G>V res. 195-220 | WT res. 197-222 | 10.3 | LH |
| 2 | 2.937 | M2 G>V res. 195-220 | WT res. 197-222 | -60 | RH |
| 3 | 2.897 | M2 G>V res. 195-220 | WT res. 197-222 | -25.1 | RH |
| 4 | 2.807 | M2 G>V res. 195-220 | WT res. 197-222 | 35 | LH |
| 5 | 2.704 | M2 G>V res. 195-220 | WT res. 197-222 | 60 | LH |
|  |  |  |  |  |  |
| 1 | 3.408 | WT res. 195-220 | WT res. 190-215 | -45.1 | RH |
| 2 | 3.383 | WT res. 195-220 | WT res. 190-215 | -5.1 | RH |
| 3 | 3.183 | WT res. 195-220 | WT res. 190-215 | 60 | LH |
| 4 | 2.934 | WT res. 195-220 | WT res. 190-215 | 20.2 | LH |
| 5 | 2.442 | WT res. 195-220 | WT res. 190-215 | 40 | LH |
|  |  |  |  |  |  |
| 1 | 3.889 | M1 G>V res. 195-220 | WT res. 190-215 | -50.1 | RH |
| 2 | 3.159 | M1 G>V res. 195-220 | WT res. 190-215 | 10.6 | LH |
| 3 | 2.851 | M1 G>V res. 195-220 | WT res. 190-215 | -15.3 | RH |
| 4 | 2.481 | M1 G>V res. 195-220 | WT res. 190-215 | 55 | LH |
|  |  |  |  |  |  |
| 1 | 2.972 | M2 G>V res. 195-220 | WT res. 190-215 | -60 | RH |
| 2 | 2.76 | M2 G>V res. 195-220 | WT res. 190-215 | 50 | LH |
| 3 | 2.568 | M2 G>V res. 195-220 | WT res. 190-215 | -25.1 | RH |
| 4 | 2.177 | M2 G>V res. 195-220 | WT res. 190-215 | 10.1 | LH |

**Figure Legends**

Supporting Information – Figure 1. **Second, Third and Fourth Ranked TMD Heterodimer Models Obtained for the HLA-DR M1 G>V Mutant in PREDDIMER.** The highest ranked models (Fig. 4) did not yield M1 or M2 packing and prevents packing of the glycine-rich faces of the two helices. The remaining models (this figure) show the same trend, whereby either the GxxxG motif in DRB1 (shown in orange) is excluded from the helix-helix interface (Rank 2-3 Models) or the M2 GxxxG motif in DRA (shown in blue) is excluded from the interface (Rank 4 Model).

Supporting Information – Figure 2. **Molecular Modelling of TM Heterodimers between I-A^k^ TM Domains using PREDDIMER.** For each sequence, the highest ranked model obtained from PREDDIMER is shown. The two GxxxG motifs in the Aαk TM domain are shown as red spheres (M1) and blue spheres (M2), and the GxxxG motif in Aβk is shown in orange. Additional residues packed at the helix-helix interface are shown as spheres, colored by element, and labelled. Residues that form part of a G>V mutation are shown in ball and stick representation. Wild type sequences yielded heterodimers stabilised by packing of either the Aαk M1 motif **(A)** or M2 motif **(B)** against the Aβk GxxxG motif. **(C)** As observed for the HLA-DR TMD models (Figure 4), mutation of the M1 motif to valine (G202V, G206V) prevents M1 pairing of the TM domains, and more broadly prevented packing of the Gly-rich faces of the two TM domains. The top ranked model is shown here, in which the Aβk GxxxG motif packs against bulky and/or polar residues in Aαk while the M1/M2 motifs are exposed to lipid. **(D)** M2-pairing of the helices is tolerant of the G202V, G206V mutation, yielding models similar to wild-type. Mutation of the M2 motif to valine (G209V, G213V) yields heterodimers very similar to wild-type, with the top ranked models illustrating both M1- **(E)** and M2- **(F)** pairing.

Supporting Information – Figure 3. **Anti-DR mAb Blocking Study.** Panel A: Cells were treated with 250 µg/ml unlabeled blocking anti-DR mAb (39-10-8 is an anti-mouse I-A^d^ mAb, included as a negative control) before addition of 1 µg/ml L243-PE or Tü36-PE. Washed cells were analyzed by flow cytometry. Shown are representative results from 1 of 3 independent experiments. Panel B: Analysis of anti-DR blocking results across 3 independent experiments. Binding of L243-PE or Tü36-PE in the absence of any inhibitor was normalized to 1.0 for each experiment and all other results reported relative to that value. Bars indicate ±1 SD from across 3 independent experiments.

Supporting Information – Figure 4. **Ratiometric Analysis of Tü36 mAb Binding to HLA-DR Mutants.** 293T cells were transfected with the indicated HLA-DR4 molecules and then stained simultaneously with L243-FITC and Tü36-PE. Dot plots show the level of staining of each population for the two mAbs (black dots represent staining of non-transfected cells). The ratio of Tü36 to L243 binding (PE:FITC fluorescence ratio, M1-paired HLA-DR:total HLA-DR) was determined on a cell-by-cell basis. Those values are compared in the histograms. The M1 G>V mutant elicits a dramatic decrease in the Tü36:L243 ratio (decreased M1 conformer expression). The DRB T140A mutation elicits a notable increase in the ratio, whereas the DRB Q149H mutant elicits a reproducible decrease. The very high Tü36:L243 ratio for the DRB T140A mutant [orange trace] is because some cells bind almost no detectable L243, but detectable amounts of Tü36. Thus, the ratio of Tü36:L243 for these cells is very high. Shown are representative results from 1 of 3 or more independent experiments (Because of differences in experiment-to-experiment transfection levels and fine instrument settings, it is difficult to compare ratios across multiple experimental runs.).

Supporting Information – Figure 5. **Amino Acid Sequence Alignment of DRB Chains Tested for Tü36 Reactivity by Single Antigen Bead (SAB) Assay.** The amino acid sequence of the mature DRB protein for each DR allele tested for Tü36 mAb reactivity by SAB assay (Fig. 6) was downloaded from the IMGT HLA database (https://www.ebi.ac.uk/ipd/imgt/hla/) and those sequences aligned in MacVector 12.7.5 using ClustalW. Alleles are ordered from highest to lowest Tü36 reactivity (top to bottom). The red line indicates the inflection point in Tü36 reactivity (see Fig. 6). The red and blue arrows below the sequences indicated residues DRB 140 and 149 (see text for details). The MacVector program does not allow colons in file names, so DRB allele names were modified (e.g., “DRB1*01:01” was modified to “DRB1*01-01”).

Supporting Information – Figure 6. **AlphaFold2 Model of Mature Full-length DR4 Protein.** The mature DR4 polypeptides were modelled in AlphaFold2 (see methods). The resulting structure is colored according to the predicted local distance difference test (pLDDT) confidence measure in the B-factor field. Highest confidence regions are colored dark blue, lowest confidence regions are colored orange, and intermediate confidence regions are yellow-light blue. The high flexibility region of DRB 105-112 is circled in the figure. DeepTMHMM was used to predict the limits of the transmembrane domains (residues shown as spheres). Position of the lipid bilayer is indicated by dashed lines.

Supporting Information – Figure 7. **Amino Acid Sequence-based Phylogeny of DRB1 Allele Families.** The amino acid sequence of the “*xx:xx:01” alleles of each indicated DRB family were used to determine the group’s phylogenetic tree using the <https://www.phylogeny.fr/> server (see methods). To the right of the obtained tree is an indication of the amino acid residues present at positions 140 and 149 of each allele (see figure 6). The text color on the allele/amino acid list generally corresponds to high (black), medium (red) or low (green) Tü36 binding (Fig. 6)
