# Supplementary figures and images for: Differential pairing of transmembrane domain GxxxG dimerization motifs defines two HLA-DR MHC class II conformers
Source: J Biol Chem. 2023 May 27;299(7):104869. doi: 10.1016/j.jbc.2023.104869 (PMC10320510; doi:10.1016/j.jbc.2023.104869)

## Supporting Information – Figure 1

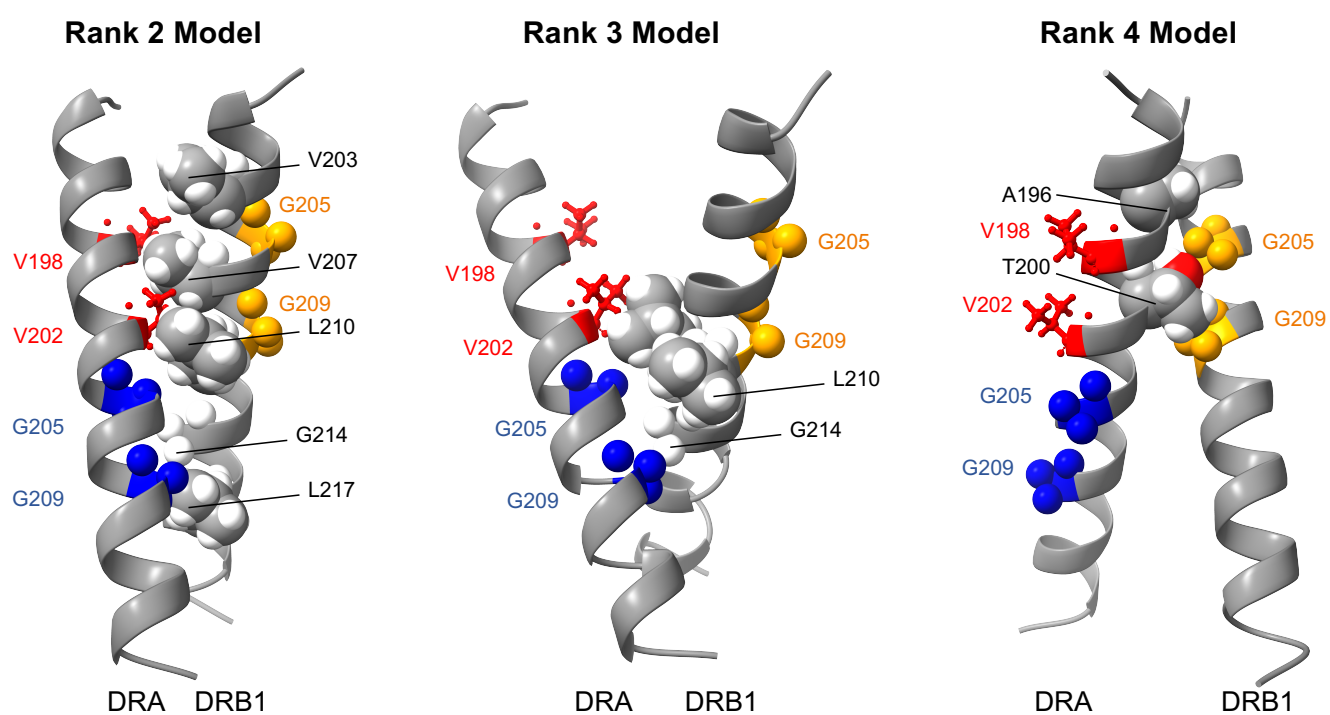

Supplement: Supplemental Figure S1 [file mmc2.pdf]

## Supporting Information – Figure 2

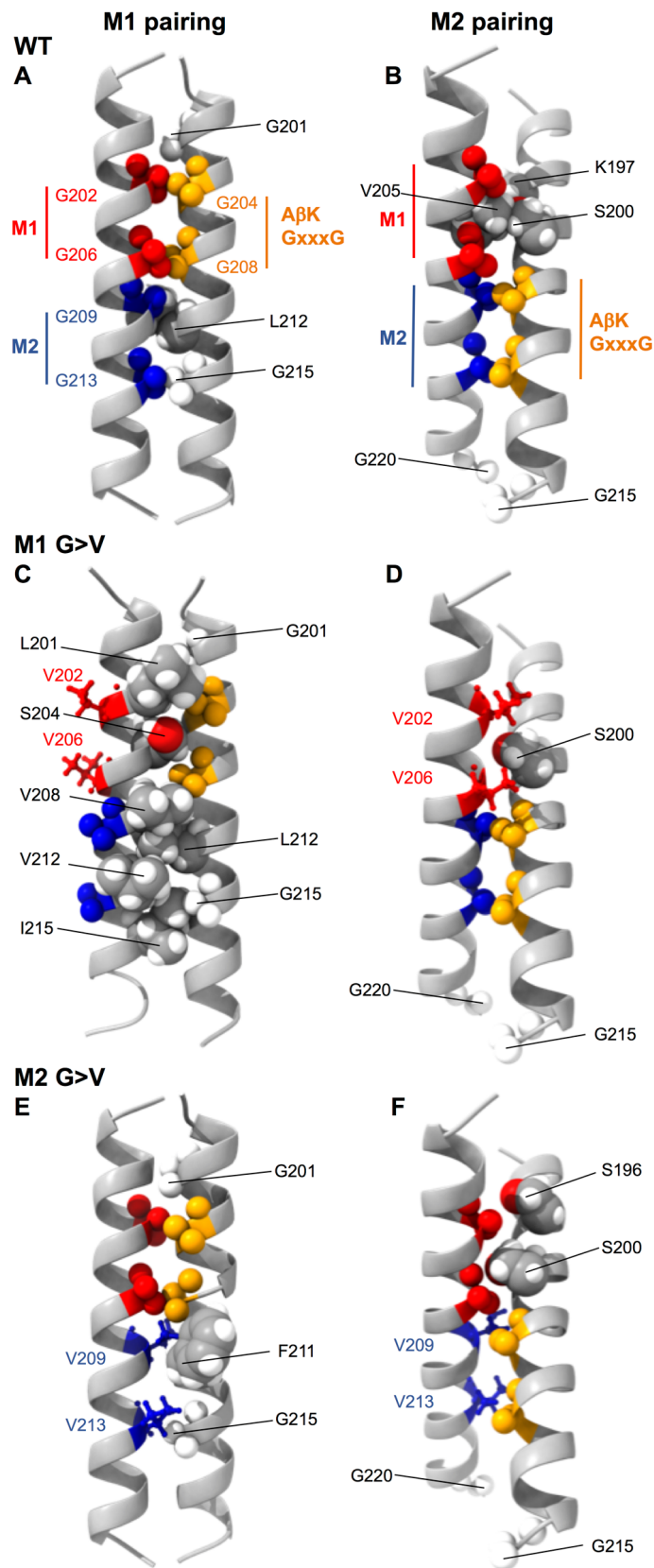

Supplement: Supplemental Figure S2 [file mmc3.pdf]

## Supporting Information – Figure 3

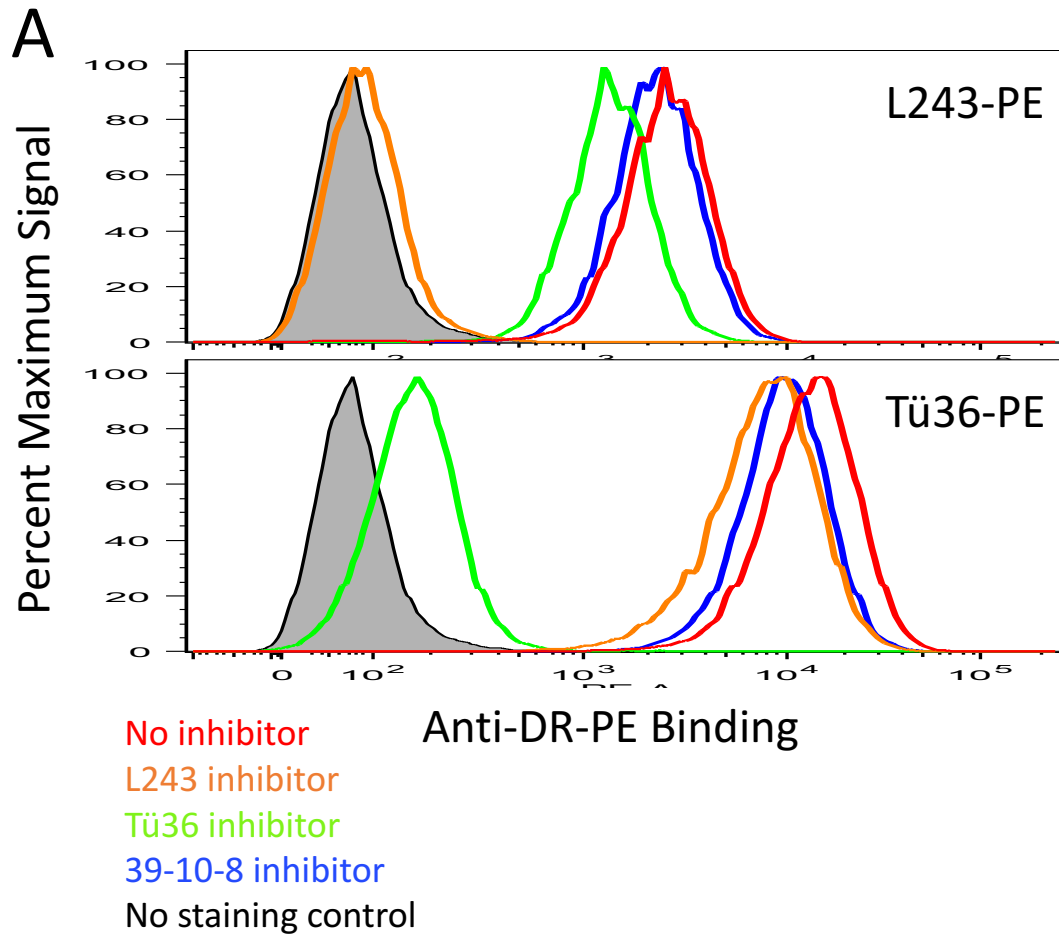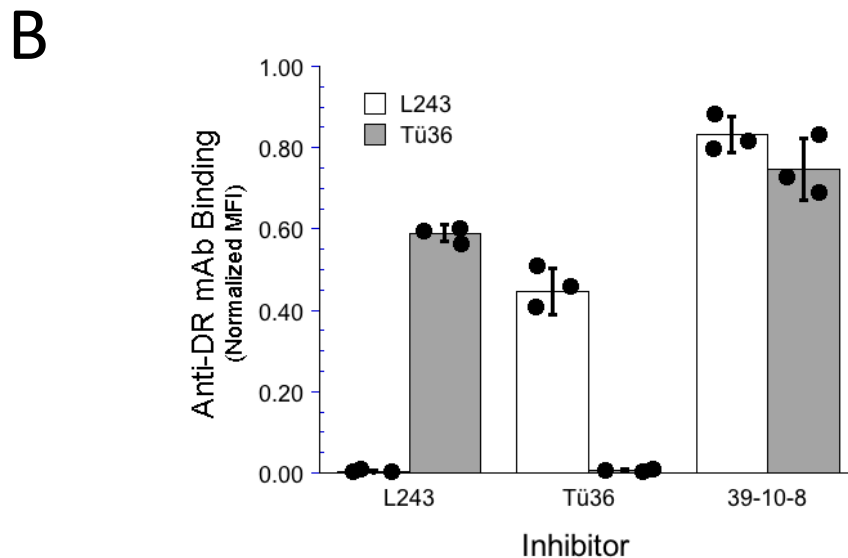

Supplement: Supplemental Figure S3 [file mmc4.pdf]

Supporting Information – Figure 4

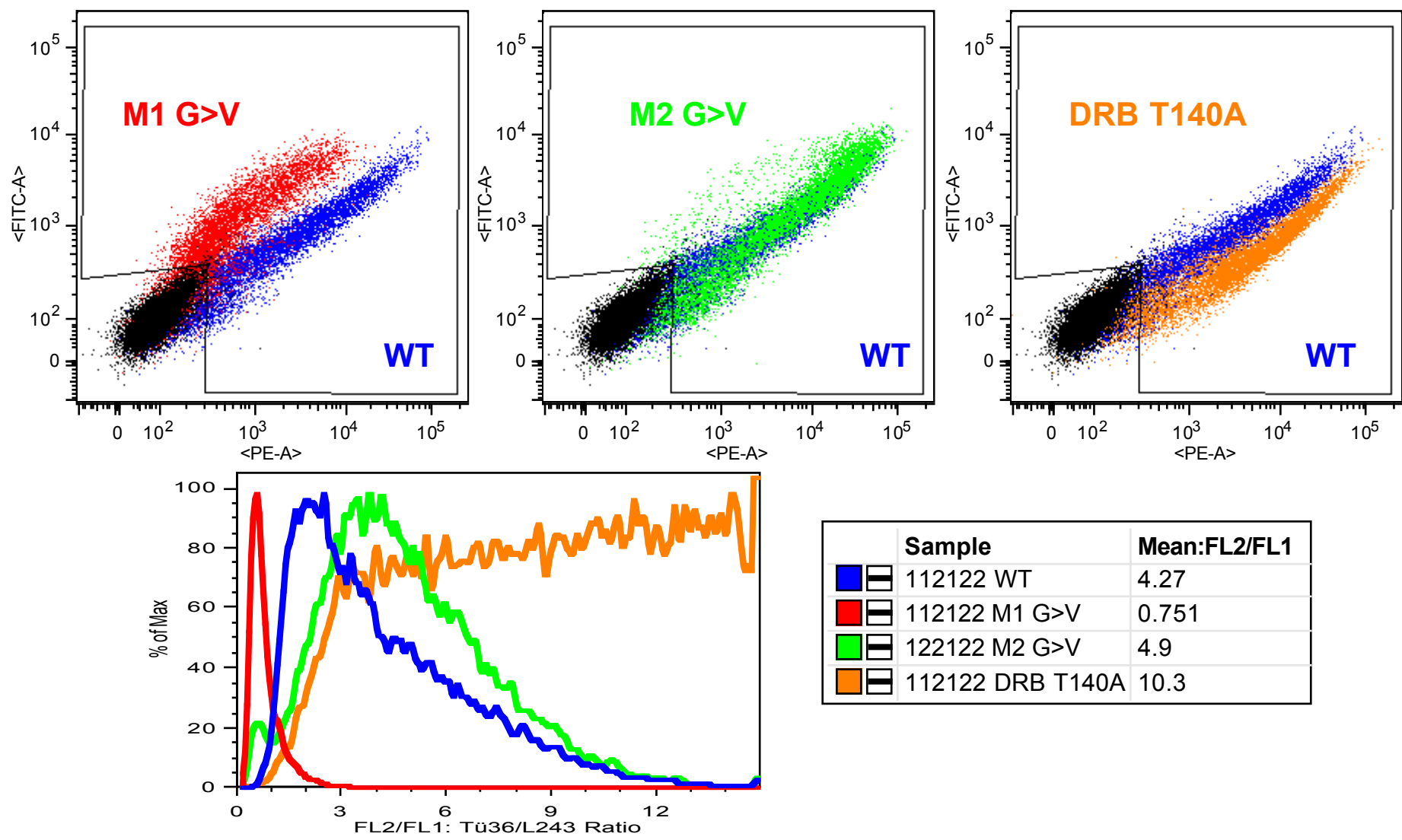

Supporting Information – Figure 4

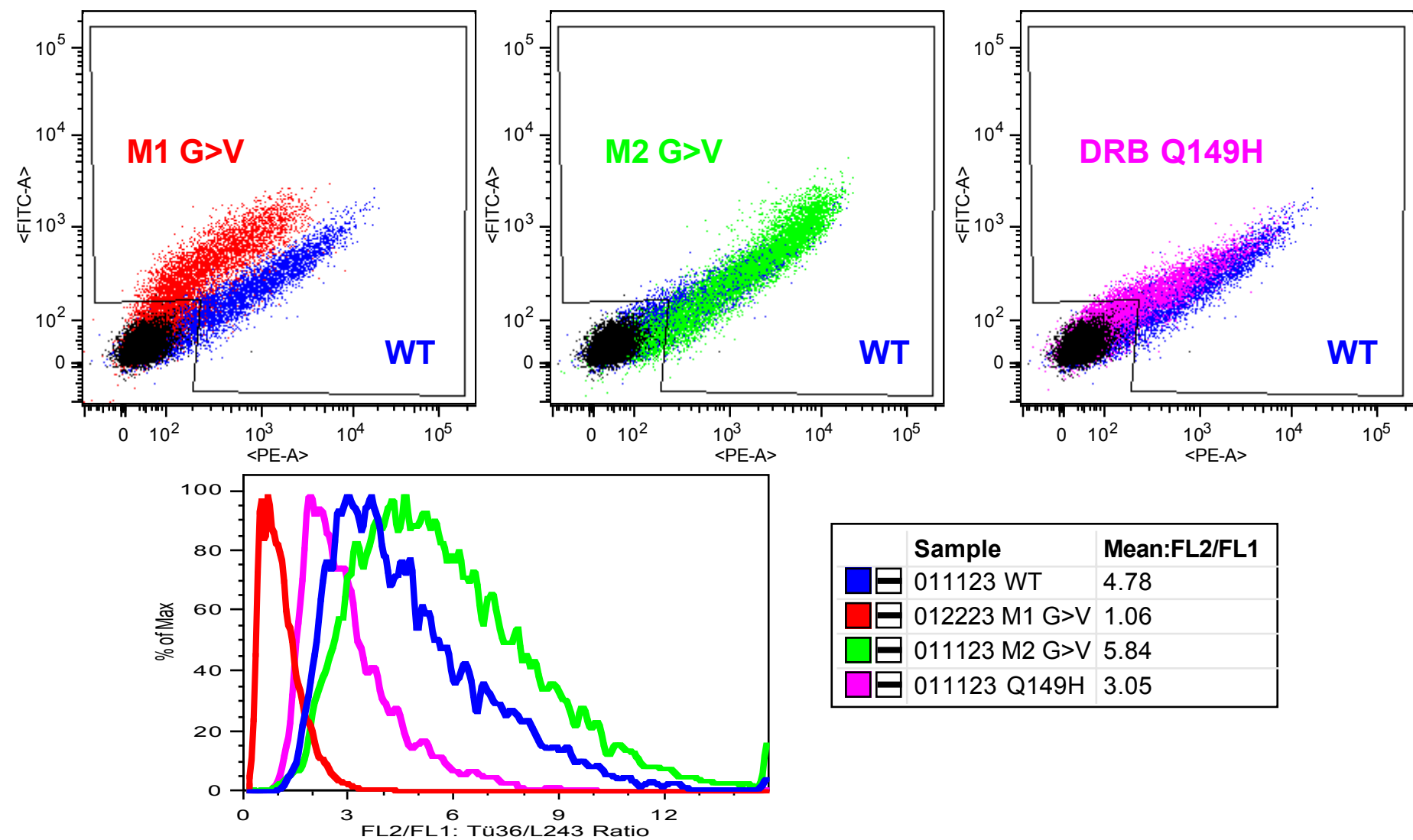

Supplement: Supplemental Figure S4 [file mmc5.pdf]

## Supporting Information Fig. 6 – AlphaFold Model of Mature Full-Length DR4 Protein

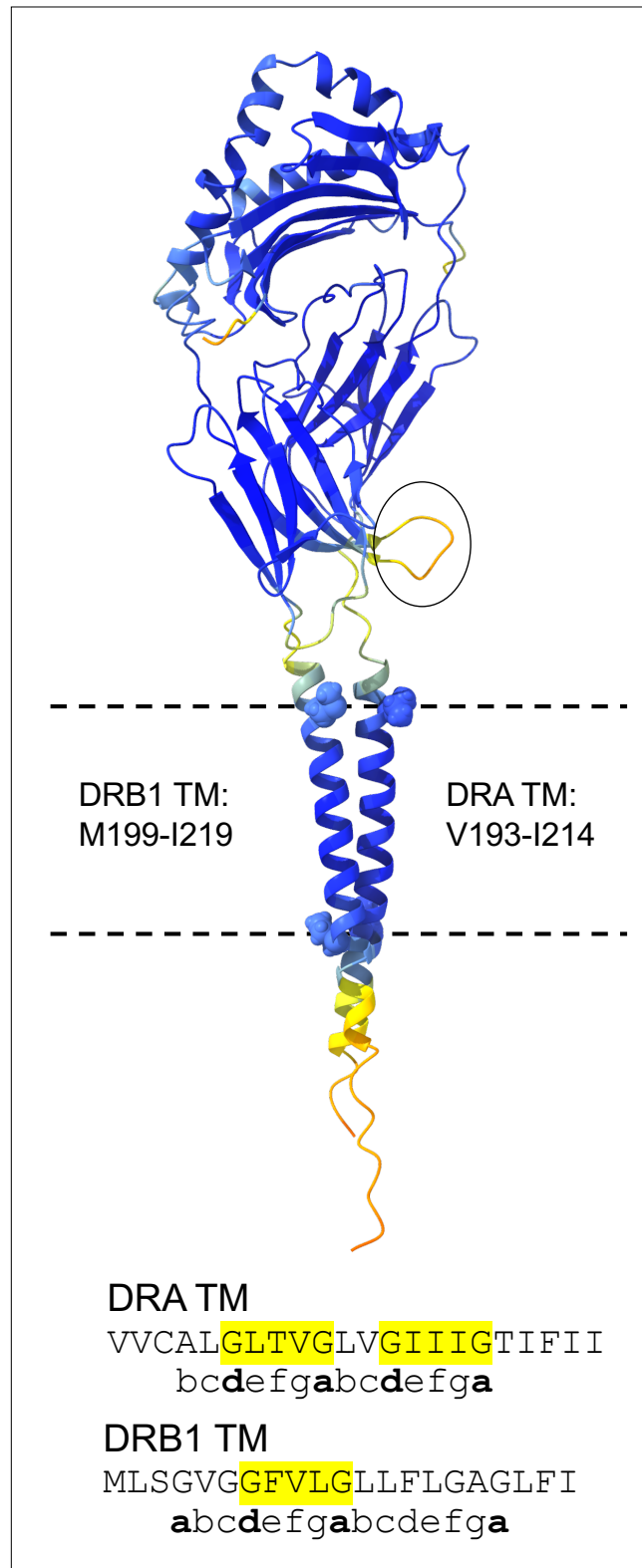

Supplement: Supplemental Figure S6 [file mmc7.pdf]

# Supporting Information – Figure 7

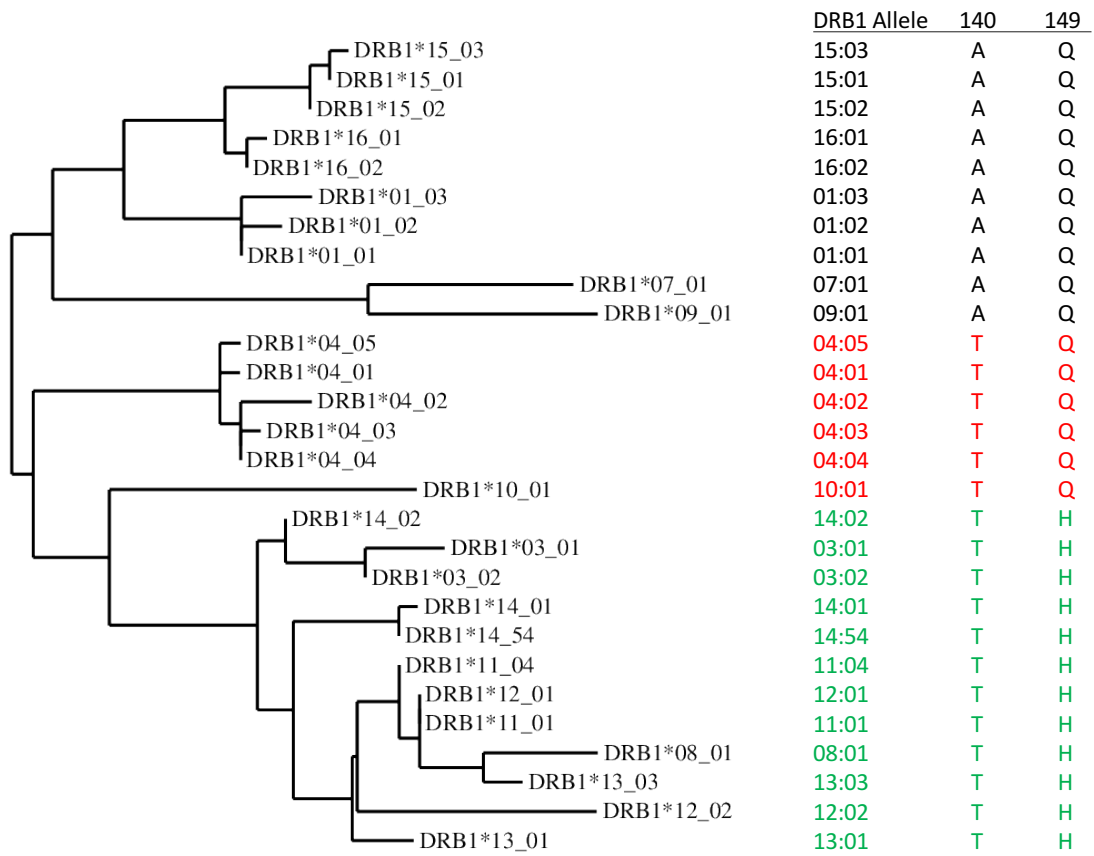

Supplement: Supplemental Figure S7 [file mmc8.pdf]
