# Supplementary material for: Differential pairing of transmembrane domain GxxxG dimerization motifs defines two HLA-DR MHC class II conformers
Source: J Biol Chem. 2023 May 27;299(7):104869. doi: 10.1016/j.jbc.2023.104869 (PMC10320510; doi:10.1016/j.jbc.2023.104869)
Supplement: Supplemental Figure S5 [file mmc6.pdf]

Supporting Information - Figure 5

	10	20	30	40	50	60	70	80
DRB1*01-01	GDTRPRFLWQ	LKFECHFFNG	TERVRLLERC	IYNQEESVRF	DSDVGEYRAV	TELGRPDAEY	WNSQKDLLEQ	RRAAVDTYCR
DRB1*01-02	GDTRPRFLWQ	LKFECHFFNG	TERVRLLERC	IYNQEESVRF	DSDVGEYRAV	TELGRPDAEY	WNSQKDLLEQ	RRAAVDTYCR
DRB1*01-03	GDTRPRFLWQ	LKFECHFFNG	TERVRLLERC	IYNQEESVRF	DSDVGEYRAV	TELGRPDAEY	WNSQKDILED	ERAAVDTYCR
DRB4*01-03	GDTQPRFLEQ	AKCECHFLNG	TERVWNLIRY	IYNQEEYARY	NSDLGEYQAV	TELGRPDAEY	WNSQKDLLER	RRAEVDTYCR
DRB4*01-01	GDTQPRFLEQ	AKCECHFLNG	TERVWNLIRY	IYNQEEYARY	NSDLGEYQAV	TELGRPDAEY	WNSQKDLLER	RRAEVDTYCR
DRB1*15-02	GDTRPRFLWQ	PKRECHFFNG	TERVRFLDRY	FYNQEESVRF	DSDVGEFRAV	TELGRPDAEY	WNSQKDILEQ	ARAAVDTYCR
DRB1*07-01	GDTQPRFLWQ	GKYKCHFFNG	TERVQFLERL	FYNQEEFVRF	DSDVGEYRAV	TELGRPVAES	WNSQKDILED	RRGQVDTVCR
DRB1*16-01	GDTRPRFLWQ	PKRECHFFNG	TERVRFLDRY	FYNQEESVRF	DSDVGEYRAV	TELGRPDAEY	WNSQKDFLED	RRAAVDTYCR
DRB1*15-03	GDTRPRFLWQ	PKRECHFFNG	TERVRFLDRH	FYNQEESVRF	DSDVGEFRAV	TELGRPDAEY	WNSQKDILEQ	ARAAVDTYCR
DRB5*02-02	GDTRPCFLQQ	DKYECHFFNG	TERVRFLHRG	IYNQEENVRF	DSDVGEYRAV	TELGRPDAEY	WNSQKDILEQ	ARAAVDTYCR
DRB1*16-02	GDTRPRFLWQ	PKRECHFFNG	TERVRFLDRY	FYNQEESVRF	DSDVGEYRAV	TELGRPDAEY	WNSQKDLLED	RRAAVDTYCR
DRB5*01-01	GDTRPRFLQQ	DKYECHFFNG	TERVRFLHRD	IYNQEEDLRF	DSDVGEYRAV	TELGRPDAEY	WNSQKDFLED	RRAAVDTYCR
DRB1*09-01	GDTQPRFLKQ	DKFECHFFNG	TERVRYLHRG	IYNQEENVRF	DSDVGEYRAV	TELGRPVAES	WNSQKDFLER	RRAEVDTVCR
DRB1*15-01	GDTRPRFLWQ	PKRECHFFNG	TERVRFLDRY	FYNQEESVRF	DSDVGEFRAV	TELGRPDAEY	WNSQKDILEQ	ARAAVDTYCR
DRB3*01-01	GDTRPRFLEL	RKSECHFFNG	TERVRYLDRY	FHNQEEFLRF	DSDVGEYRAV	TELGRPVAES	WNSQKDLLEQ	KRGRVDNYCR
DRB3*02-02	GDTRPRFLEL	LKSECHFFNG	TERVRFLERH	FHNQEEYARF	DSDVGEYRAV	RELGRPDAEY	WNSQKDLLEQ	KRGQVDNYCR
DRB1*04-01	GDTRPRFLEQ	VKHECHFFNG	TERVRFLDRY	FYHQEEYVRF	DSDVGEYRAV	TELGRPDAEY	WNSQKDLLEQ	KRAAVDTYCR
DRB1*04-03	GDTRPRFLEQ	VKHECHFFNG	TERVRFLDRY	FYHQEEYVRF	DSDVGEYRAV	TELGRPDAEY	WNSQKDLLEQ	RRAEVDTYCR
DRB1*04-04	GDTRPRFLEQ	VKHECHFFNG	TERVRFLDRY	FYHQEEYVRF	DSDVGEYRAV	TELGRPDAEY	WNSQKDLLEQ	RRAAVDTYCR
DRB1*04-02	GDTRPRFLEQ	VKHECHFFNG	TERVRFLDRY	FYHQEEYVRF	DSDVGEYRAV	TELGRPDAEY	WNSQKDILED	ERAAVDTYCR
DRB1*13-01	GDTRPRFLEY	STSECHFFNG	TERVRFLDRY	FHNQEENVRF	DSDVGEFRAV	TELGRPDAEY	WNSQKDILED	ERAAVDTYCR
DRB1*13-03	GDTRPRFLEY	STSECHFFNG	TERVRFLDRY	FYNQEEYVRF	DSDVGEYRAV	TELGRPSAEY	WNSQKDILED	KRAAVDTYCR
DRB1*03-01	GDTRPRFLEY	STSECHFFNG	TERVRYLDRY	FHNQEENVRF	DSDVGEFRAV	TELGRPDAEY	WNSQKDLLEQ	KRGRVDNYCR
DRB1*14-01	GDTRPRFLEY	STSECHFFNG	TERVRFLDRY	FHNQEEFVRF	DSDVGEYRAV	TELGRPAAEH	WNSQKDLLER	RRAEVDTYCR
DRB1*12-01	GDTRPRFLEY	STGECYFFNG	TERVRLLERH	FHNQEELLRF	DSDVGEFRAV	TELGRPVAES	WNSQKDILED	RRAAVDTYCR
DRB1*04-05	GDTRPRFLEQ	VKHECHFFNG	TERVRFLDRY	FYHQEEYVRF	DSDVGEYRAV	TELGRPSAEY	WNSQKDLLEQ	RRAAVDTYCR
DRB1*08-01	GDTRPRFLEY	STGECYFFNG	TERVRFLDRY	FYNQEEYVRF	DSDVGEYRAV	TELGRPSAEY	WNSQKDFLED	RRALVDTYCR
DRB1*12-02	GDTRPRFLEY	STGECYFFNG	TERVRLLERH	FHNQEELLRF	DSDVGEFRAV	TELGRPVAES	WNSQKDFLED	RRAAVDTYCR
DRB1*14-54	GDTRPRFLEY	STSECHFFNG	TERVRFLDRY	FHNQEEFVRF	DSDVGEYRAV	TELGRPAAEH	WNSQKDLLER	RRAEVDTYCR
DRB1*11-01	GDTRPRFLEY	STSECHFFNG	TERVRFLDRY	FYNQEEYVRF	DSDVGEFRAV	TELGRPDEEY	WNSQKDFLED	RRAAVDTYCR
DRB3*03-01	GDTRPRFLEL	LKSECHFFNG	TERVRFLERY	FHNQEEFVRF	DSDVGEYRAV	TELGRPVAES	WNSQKDLLEQ	KRGQVDNYCR
DRB1*14-02	GDTRPRFLEY	STSECHFFNG	TERVRFLERY	FHNQEENVRF	DSDVGEYRAV	TELGRPDAEY	WNSQKDLLEQ	RRAAVDTYCR
DRB1*11-04	GDTRPRFLEY	STSECHFFNG	TERVRFLDRY	FYNQEEYVRF	DSDVGEFRAV	TELGRPDEEY	WNSQKDFLED	RRAAVDTYCR
DRB1*03-02	GDTRPRFLEY	STSECHFFNG	TERVRFLERY	FHNQEENVRF	DSDVGEYRAV	TELGRPDAEY	WNSQKDLLEQ	KRGRVDNYCR
DRB1*10-01	GDTRPRFLEE	VKFECHFFNG	TERVRLLERR	VHNQEEYARY	DSDVGEYRAV	TELGRPDAEY	WNSQKDLLER	RRAAVDTYCR

	90	100	110	120	130	140	150	160
DRB1*01-01	HNYGVGESFT	VQRRVEPKVT	VYPSKTQPLQ	HHNLLVCSVS	GFYPGSIEVR	WFRNGQEEKA	GVVSTGLIQN	GDWTFQTLVM
DRB1*01-02	HNYGAVESFT	VQRRVEPKVT	VYPSKTQPLQ	HHNLLVCSVS	GFYPGSIEVR	WFRNGQEEKA	GVVSTGLIQN	GDWTFQTLVM
DRB1*01-03	HNYGVGESFT	VQRRVEPKVT	VYPSKTQPLQ	HHNLLVCSVS	GFYPGSIEVR	WFRNGQEEKA	GVVSTGLIQN	GDWTFQTLVM
DRB4*01-03	YNYGVVESFT	VQRRVQPKVT	VYPSKTQPLQ	HHNLLVCSVN	GFYPGSIEVR	WFRNGQEEKA	GVVSTGLIQN	GDWTFQTLVM
DRB4*01-01	YNYGVVESFT	VQRRVQPKVT	VYPSKTQPLQ	HHNLLVCSVN	GFYPGSIEVR	WFRNSQEEKA	GVVSTGLIQN	GDWTFQTLVM
DRB1*15-02	HNYGVGESFT	VQRRVQPKVT	VYPSKTQPLQ	HHNLLVCSVS	GFYPGSIEVR	WFLNGQEEKA	GMVSTGLIQN	GDWTFQTLVM
DRB1*07-01	HNYGVGESFT	VQRRVHPEVT	VYPAKTQPLQ	HHNLLVCSVS	GFYPGSIEVR	WFRNGQEEKA	GVVSTGLIQN	GDWTFQTLVM
DRB1*16-01	HNYGVGESFT	VQRRVQPKVT	VYPSKTQPLQ	HHNLLVCSVS	GFYPGSIEVR	WFLNGQEEKA	GMVSTGLIQN	GDWTFQTLVM
DRB1*15-03	HNYGVVESFT	VQRRVQPKVT	VYPSKTQPLQ	HHNLLVCSVS	GFYPGSIEVR	WFLNGQEEKA	GMVSTGLIQN	GDWTFQTLVM
DRB5*02-02	HNYGAVESFT	VQRRVEPKVT	VYPARTQTLQ	HHNLLVCSVN	GFYPGSIEVR	WFRNGQEEKA	GVVSTGLIQN	GDWTFQILVM
DRB1*16-02	HNYGVGESFT	VQRRVQPKVT	VYPSKTQPLQ	HHNLLVCSVS	GFYPGSIEVR	WFLNGQEEKA	GMVSTGLIQN	GDWTFQTLVM
DRB5*01-01	HNYGVGESFT	VQRRVEPKVT	VYPARTQTLQ	HHNLLVCSVN	GFYPGSIEVR	WFRNSQEEKA	GVVSTGLIQN	GDWTFQTLVM
DRB1*09-01	HNYGVGESFT	VQRRVHPEVT	VYPAKTQPLQ	HHNLLVCSVS	GFYPGSIEVR	WFRNGQEEKA	GVVSTGLIQN	GDWTFQTLVM
DRB1*15-01	HNYGVVESFT	VQRRVQPKVT	VYPSKTQPLQ	HHNLLVCSVS	GFYPGSIEVR	WFLNGQEEKA	GMVSTGLIQN	GDWTFQTLVM
DRB3*01-01	HNYGVGESFT	VQRRVHPQVT	VYPAKTQPLQ	HHNLLVCSVS	GFYPGSIEVR	WFRNGQEEKA	GVVSTGLIQN	GDWTFQTLVM
DRB3*02-02	HNYGVGESFT	VQRRVHPQVT	VYPAKTQPLQ	HHNLLVCSVS	GFYPGSIEVR	WFRNGQEEKA	GVVSTGLIQN	GDWTFQTLVM
DRB1*04-01	HNYGVGESFT	VQRRVYPEVT	VYPAKTQPLQ	HHNLLVCSVN	GFYPGSIEVR	WFRNGQEEKT	GVVSTGLIQN	GDWTFQTLVM
DRB1*04-03	HNYGVVESFT	VQRRVYPEVT	VYPAKTQPLQ	HHNLLVCSVN	GFYPGSIEVR	WFRNGQEEKT	GVVSTGLIQN	GDWTFQTLVM
DRB1*04-04	HNYGVVESFT	VQRRVYPEVT	VYPAKTQPLQ	HHNLLVCSVN	GFYPGSIEVR	WFRNGQEEKT	GVVSTGLIQN	GDWTFQTLVM
DRB1*04-02	HNYGVVESFT	VQRRVYPEVT	VYPAKTQPLQ	HHNLLVCSVN	GFYPGSIEVR	WFRNGQEEKT	GVVSTGLIQN	GDWTFQTLVM
DRB1*13-01	HNYGVVESFT	VQRRVHPKVT	VYPSKTQPLQ	HHNLLVCSVS	GFYPGSIEVR	WFRNGQEEKT	GVVSTGLIHN	GDWTFQTLVM
DRB1*13-03	HNYGVGESFT	VQRRVHPKVT	VYPSKTQPLQ	HHNLLVCSVS	GFYPGSIEVR	WFRNGQEEKT	GVVSTGLIHN	GDWTFQTLVM
DRB1*03-01	HNYGVVESFT	VQRRVHPKVT	VYPSKTQPLQ	HHNLLVCSVS	GFYPGSIEVR	WFRNGQEEKT	GVVSTGLIHN	GDWTFQTLVM
DRB1*14-01	HNYGVVESFT	VQRRVHPKVT	VYPSKTQPLQ	HYNLLVCSVS	GFYPGSIEVR	WFRNGQEEKT	GVVSTGLIHN	GDWTFQTLVM
DRB1*12-01	HNYGAVESFT	VQRRVHPKVT	VYPSKTQPLQ	HHNLLVCSVS	GFYPGSIEVR	WFRNGQEEKT	GVVSTGLIHN	GDWTFQTLVM
DRB1*04-05	HNYGVGESFT	VQRRVYPEVT	VYPAKTQPLQ	HHNLLVCSVN	GFYPGSIEVR	WFRNGQEEKT	GVVSTGLIQN	GDWTFQTLVM
DRB1*08-01	HNYGVGESFT	VQRRVHPKVT	VYPSKTQPLQ	HHNLLVCSVS	GFYPGSIEVR	WFRNGQEEKT	GVVSTGLIHN	GDWTFQTLVM
DRB1*12-02	HNYGAVESFT	VQRRVHPKVT	VYPSKTQPLQ	HHNLLVCSVS	GFYPGSIEVR	WFRNGQEEKT	GVVSTGLIHN	GDWTFQTLVM
DRB1*14-54	HNYGVVESFT	VQRRVHPKVT	VYPSKTQPLQ	HHNLLVCSVS	GFYPGSIEVR	WFRNGQEEKT	GVVSTGLIHN	GDWTFQTLVM
DRB1*11-01	HNYGVGESFT	VQRRVHPKVT	VYPSKTQPLQ	HHNLLVCSVS	GFYPGSIEVR	WFRNGQEEKT	GVVSTGLIHN	GDWTFQTLVM
DRB3*03-01	HNYGVVESFT	VQRRVHPQVT	VYPAKTQPLQ	HHNLLVCSVS	GFYPGSIEVR	WFRNGQEEKT	GVVSTGLIHN	GDWTFQTLVM
DRB1*14-02	HNYGVGESFT	VQRRVHPKVT	VYPSKTQPLQ	HHNLLVCSVS	GFYPGSIEVR	WFRNGQEEKT	GVVSTGLIHN	GDWTFQTLVM
DRB1*11-04	HNYGVVESFT	VQRRVHPKVT	VYPSKTQPLQ	HHNLLVCSVS	GFYPGSIEVR	WFRNGQEEKT	GVVSTGLIHN	GDWTFQTLVM
DRB1*03-02	HNYGVGESFT	VQRRVHPKVT	VYPSKTQPLQ	HHNLLVCSVS	GFYPGSIEVR	WFRNGQEEKT	GVVSTGLIHN	GDWTFQTLVM
DRB1*10-01	HNYGVGESFT	VQRRVQPKVT	VYPSKTQPLQ	HHNLLVCSVN	GFYPGSIEVR	WFRNGQEEKT	GVVSTGLIQN	GDWTFQTLVM

	170	180	190	200	210	220	230	
**DRB1*01-01**	LETVPRSGEV	YTCQVEHPSV	TSPLTVEWRA	RSESAQSKML	SGVGGFVLGL	LFLGAGLFIY	FRNQKGHSGL	QPTGFLS
**DRB1*01-02**	LETVPRSGEV	YTCQVEHPSV	TSPLTVEWRA	RSESAQSKML	SGVGGFVLGL	LFLGAGLFIY	FRNQKGHSGL	QPTGFLS
**DRB1*01-03**	LETVPRSGEV	YTCQVEHPSV	TSPLTVEWRA	RSESAQSKML	SGVGGFVLGL	LFLGAGLFIY	FRNQKGHSGL	QPTGFLS
**DRB4*01-03**	LETVPRSGEV	YTCQVEHPSM	MSPLTVQWSA	RSESAQSKML	SGVGGFVLGL	LFLGTGLFIY	FRNQKGHSGL	QPTGLLS
**DRB4*01-01**	LETVPRSGEV	YTCQVEHPSM	MSPLTVQWSA	RSESAQSKML	SGVGGFVLGL	LFLGTGLFIY	FRNQKGHSGL	QPTGLLS
**DRB1*15-02**	LETVPRSGEV	YTCQVEHPSV	TSPLTVEWRA	RSESAQSKML	SGVGGFVLGL	LFLGAGLFIY	FRNQKGHSGL	QPTGFLS
**DRB1*07-01**	LETVPRSGEV	YTCQVEHPSV	MSPLTVEWRA	RSESAQSKML	SGVGGFVLGL	LFLGAGLFIY	FRNQKGHSGL	QPTGFLS
**DRB1*16-01**	LETVPRSGEV	YTCQVEHPSV	TSPLTVEWRA	RSESAQSKML	SGVGGFVLGL	LFLGAGLFIY	FRNQKGHSGL	QPTGFLS
**DRB1*15-03**	LETVPRSGEV	YTCQVEHPSV	TSPLTVEWRA	RSESAQSKML	SGVGGFVLGL	LFLGAGLFIY	FRNQKGHSGL	QPTGFLS
**DRB5*02-02**	LETVPRSGEV	YTCQVEHPSV	TSPLTVEWRA	QSESAQSKML	SGIGGFVLGL	LFLGAGLFIY	FKNQKGHSGL	HPTGLVS
**DRB1*16-02**	LETVPRSGEV	YTCQVEHPSV	TSPLTVEWRA	RSESAQSKML	SGVGGFVLGL	LFLGAGLFIY	FRNQKGHSGL	QPTGFLS
**DRB5*01-01**	LETVPRSGEV	YTCQVEHPSV	TSPLTVEWRA	QSESAQSKML	SGVGGFVLGL	LFLGAGLFIY	FKNQKGHSGL	HPTGLVS
**DRB1*09-01**	LETVPRSGEV	YTCQVEHPSV	MSPLTVEWRA	RSESAQSKML	SGVGGFVLGL	LFLGAGLFIY	FRNQKGHSGL	QPTGFLS
**DRB1*15-01**	LETVPRSGEV	YTCQVEHPSV	TSPLTVEWRA	RSESAQSKML	SGVGGFVLGL	LFLGAGLFIY	FRNQKGHSGL	QPTGFLS
**DRB3*01-01**	LETVPRSGEV	YTCQVEHPSV	TSALTVEWRA	RSESAQSKML	SGVGGFVLGL	LFLGAGLFIY	FRNQKGHSGL	QPTGFLS
**DRB3*02-02**	LETVPRSGEV	YTCQVEHPSV	TSPLTVEWSA	RSESAQSKML	SGVGGFVLGL	LFLGAGLFIY	FRNQKGHSGL	QPTGFLS
**DRB1*04-01**	LETVPRSGEV	YTCQVEHPSL	TSPLTVEWRA	RSESAQSKML	SGVGGFVLGL	LFLGAGLFIY	FRNQKGHSGL	QPTGFLS
**DRB1*04-03**	LETVPRSGEV	YTCQVEHPSL	TSPLTVEWRA	RSESAQSKML	SGVGGFVLGL	LFLGAGLFIY	FRNQKGHSGL	QPTGFLS
**DRB1*04-04**	LETVPRSGEV	YTCQVEHPSL	TSPLTVEWRA	RSESAQSKML	SGVGGFVLGL	LFLGAGLFIY	FRNQKGHSGL	QPTGFLS
**DRB1*04-02**	LETVPRSGEV	YTCQVEHPSL	TSPLTVEWRA	RSESAQSKML	SGVGGFVLGL	LFLGAGLFIY	FRNQKGHSGL	QPTGFLS
**DRB1*13-01**	LETVPRSGEV	YTCQVEHPSV	TSPLTVEWRA	RSESAQSKML	SGVGGFVLGL	LFLGAGLFIY	FRNQKGHSGL	QPRGFLS
**DRB1*13-03**	LETVPRSGEV	YTCQVEHPSV	TSPLTVEWRA	RSESAQSKML	SGVGGFVLGL	LFLGAGLFIY	FRNQKGHSGL	QPRGFLS
**DRB1*03-01**	LETVPRSGEV	YTCQVEHPSV	TSPLTVEWRA	RSESAQSKML	SGVGGFVLGL	LFLGAGLFIY	FRNQKGHSGL	QPRGFLS
**DRB1*14-01**	LETVPRSGEV	YTCQVEHPSV	TSPLTVEWRA	RSESAQSKML	SGVGGFVLGL	LFLGAGLFIY	FRNQKGHSGL	QPRGFLS
**DRB1*12-01**	LETVPRSGEV	YTCQVEHPSV	TSPLTVEWRA	RSESAQSKML	SGVGGFVLGL	LFLGAGLFIY	FRNQKGHSGL	QPRGFLS
**DRB1*04-05**	LETVPRSGEV	YTCQVEHPSL	TSPLTVEWRA	RSESAQSKML	SGVGGFVLGL	LFLGAGLFIY	FRNQKGHSGL	QPTGFLS
**DRB1*08-01**	LETVPRSGEV	YTCQVEHPSV	TSPLTVEWSA	RSESAQSKML	SGVGGFVLGL	LFLGAGLFIY	FRNQKGHSGL	QPTGFLS
**DRB1*12-02**	LETVPRSGEV	YTCQVEHPSV	TSPLTVEWRA	RSESAQSKML	SGVGGFVLGL	LFLGAGLFIY	FRNQKGHSGL	QPRGFLS
**DRB1*14-54**	LETVPRSGEV	YTCQVEHPSV	TSPLTVEWRA	RSESAQSKML	SGVGGFVLGL	LFLGAGLFIY	FRNQKGHSGL	QPRGFLS
**DRB1*11-01**	LETVPRSGEV	YTCQVEHPSV	TSPLTVEWRA	RSESAQSKML	SGVGGFVLGL	LFLGAGLFIY	FRNQKGHSGL	QPRGFLS
**DRB3*03-01**	LETVPRSGEV	YTCQVEHPSV	TSPLTVEWRA	RSESAQSKML	SGVGGFVLGL	LFLGAGLFIY	FRNQKGHSGL	QPTGFLS
**DRB1*14-02**	LETVPRSGEV	YTCQVEHPSV	TSPLTVEWRA	RSESAQSKML	SGVGGFVLGL	LFLGAGLFIY	FRNQKGHSGL	QPRGFLS
**DRB1*11-04**	LETVPRSGEV	YTCQVEHPSV	TSPLTVEWRA	RSESAQSKML	SGVGGFVLGL	LFLGAGLFIY	FRNQKGHSGL	QPRGFLS
**DRB1*03-02**	LETVPRSGEV	YTCQVEHPSV	TSPLTVEWRA	RSESAQSKML	SGVGGFVLGL	LFLGAGLFIY	FRNQKGHSGL	QPRGFLS
**DRB1*10-01**	LETVPQSGEV	YTCQVEHPSV	MSPLTVEWRA	RSESAQSKML	SGVGGFVLGL	LFLGAGLFIY	FRNQKGHSGL	PPTGFLS
